# Expression of Keratin-1 Predicts Recurrence and Treatment Response in Advanced Laryngeal Cancer: A Potential Therapeutic Target

**DOI:** 10.3390/curroncol32090520

**Published:** 2025-09-17

**Authors:** Eun Kyung Jung, S M Abdus Salam, Hye-Bin Jang, Joo Yeon Koo, Eshrat Jahan, Sun-Ae Kim, Ji Young Lee, Kyung-Hwa Lee, Tae Mi Yoon

**Affiliations:** 1 Department of Otolaryngology-Head and Neck Surgery, Chonnam National University Medical School and Hwasun Hospital, Gwangju 58128, Republic of Korea; 2 Department of Pathology, Chonnam National University Medical School and Hwasun Hospital, Gwangju 58128, Republic of Korea; 3 Department of Biomedical Sciences, Chonnam National University Medical School and Hwasun Hospital, Gwangju 58128, Republic of Korea; 4 Department of BioMedical Sciences Graduate Program (BMSGP), Chonnam National University Hwasun Hospital and Medical School, Gwangju 58128, Republic of Korea

**Keywords:** keratin-1, laryngeal cancer, recurrence, molecular targeted therapy, radioresistance

## Abstract

**Simple Summary:**

Advanced laryngeal cancer is challenging to treat, and overall survival rates have not been improved compared to advancements in treatment methods. The high rate of disease recurrence in advanced laryngeal cancer contributes to poor outcomes. This study investigates the role of Keratin-1 (KRT1), identified through RNA sequencing as a potential target molecule for laryngeal cancer, in the tumor progression and chemoradioresistance of laryngeal cancer cells. These findings suggest that KRT1 plays a critical role in the progression and chemoradioresistance of laryngeal cancer and may serve as a promising therapeutic target. Although these results are preliminary, we propose that KRT1 may function as an innovative biomarker for predicting prognosis and aid in patient selection for concurrent chemoradiotherapy for laryngeal cancer treatment.

**Abstract:**

The survival rate of patients with advanced laryngeal cancer has not substantially improved over time. RNA sequencing analysis identified Keratin-1 (KRT1) as a gene potentially associated with cancer recurrence. This study investigated the association between KRT1 expression and recurrence in advanced laryngeal cancer. RNA sequencing was performed to identify candidate genes associated with recurrence. The effects of KRT1 expression on clinical outcomes were evaluated in patients with laryngeal cancer. Multiple experimental techniques were utilized. RNA sequencing of patient samples demonstrated higher KRT1 gene expression in the recurrence group than in non-recurrent cases. Patients with KRT1-positive immunostaining exhibited trends of worse overall survival (OS) and recurrence-free survival (RFS). In vitro studies showed that KRT1 knockdown suppressed tumor cell invasion, cell migration, and expression of epithelial–mesenchymal transition (EMT)-related genes in human head and neck squamous cell carcinoma (HNSCC) cell lines. KRT1 knockdown enhanced tumor cell apoptosis and exhibited synergistic effects with conventional radiation and chemotherapy treatments. KRT1 may serve as a biomarker for predicting advanced laryngeal cancer recurrence and assist with selecting patients to receive concurrent chemoradiotherapy (CCRT). Further molecular investigations are warranted to determine its effects, but KRT1 has potential as a therapeutic target.

## 1. Introduction

Advanced laryngeal cancer, including stages III and IV, can be treated with either extensive surgery or chemoradiation according to the National Comprehensive Cancer Network (NCCN) guidelines (www.NCCN.org). The combination of chemoradiation has enabled a large proportion of patients to preserve their organ function [1,2]. In terms of clinical outcomes such as morbidity and organ preservation, chemoradiation shows better results compared to extensive surgery, such as total laryngectomy [3]. However, survival rates for advanced laryngeal cancer have not significantly improved due to high rates of disease relapse, leading to ongoing controversy regarding optimal treatment selection [4].

NCCN guidelines recommend either surgery or chemoradiation, and various clinical factors, including patient preferences, risk of functional impairments such as voice loss, aspiration, and breathing difficulties, are considered when selecting a treatment strategy. Because these treatment modalities have comparable overall survival rates but markedly different impacts on quality of life, the clinical challenge lies not only in identifying general prognostic markers but in developing predictive biomarkers that can inform the optimal choice between extensive surgery and chemoradiation. In this context, we intentionally focused our study on a homogeneous cohort of patients treated with definitive chemoradiotherapy (CRT) to investigate which gene expression can serve as a predictive biomarker for CRT response. We hypothesized that a better understanding of molecular markers associated with treatment resistance could help stratify patients who are more or less likely to benefit from organ-preserving treatment and guide the selection of initial therapy accordingly.

Identification of novel therapeutic targets is essential to improve patient outcomes. RNA sequencing has been widely utilized in cancer research to identify predictive biomarkers associated with disease prognosis and therapeutic response [5]. Keratin-1 (KRT1), a member of the type I keratin gene family, has been reported to regulate kinase activity and participate in angiogenesis, fibrinolysis, and oxidative stress [6]. A few studies have indicated KRT1 involvement in premalignant and malignant oral lesions [7,8], but its role in laryngeal cancer remains unexplored. Interestingly, in a cisplatin-resistant human ovarian cancer cell line, KRT1 showed significant differential expression in proteomic analysis [9], and another study revealed that KRT1 may be associated with chemotherapy sensitivity in nasopharyngeal cancer [10].

This single-center study investigated the association between KRT1 expression and recurrence in advanced laryngeal cancer and then explored its potential as a therapeutic target. KRT1 expression in advanced laryngeal cancer specimens was analyzed, and its associations with clinicopathological features and survival outcomes were evaluated. Additionally, in vitro studies were conducted to assess the effects of KRT1 knockdown on cell invasion, migration, epithelial–mesenchymal transition (EMT), and apoptosis in head and neck squamous cell carcinoma (HNSCC) cell lines. The effects of KRT1 knockdown on conventional radiation and chemotherapy were also examined.

Findings from this study suggest that KRT1 contributes to laryngeal cancer recurrence and resistance to chemoradiotherapy. Thus, KRT1 has potential not only as a therapeutic target but also as a predictive biomarker to guide initial treatment selection between surgery and chemoradiation in patients with advanced laryngeal cancer.

## 2. Materials and Methods

### 2.1. Patients and Tumor Specimens

Medical records were retrospectively reviewed for patients who underwent definitive concurrent chemoradiotherapy (CCRT) for advanced laryngeal cancer at Chonnam National University Hwasun Hospital between 2015 and 2018. Advanced laryngeal cancer was defined as Stage III or IV disease according to the 7th edition of the American Joint Committee on Cancer staging system, excluding T1Nany patients [11]. This study included patients who received definitive CCRT with radiation doses exceeding 6000 cGy and excluded those who had undergone prior total laryngectomy. Only patients with known survival or recurrence status for at least 2 years were classified as non-recurrent cases. Overall survival was calculated from treatment initiation to death or last follow-up. Recurrence-free survival was measured from the start of treatment to the first recurrence or death.

Hematoxylin and eosin-stained tissue slides were retrospectively reviewed; sample selection was based on tissue availability. Representative formalin-fixed paraffin-embedded (FFPE) tissue blocks were selected for analysis. The RNA sequencing cohort comprised 32 FFPE tissue samples, including 11 recurrent and 21 non-recurrent tumor specimens that passed quality control testing. For immunohistochemistry analysis, the sample set was expanded to encompass patients diagnosed with advanced laryngeal cancer between 2011 and 2021, using the same clinical criteria. After clinical information and tissue availability had been reviewed, an additional 62 cases were selected. Three cases had no residual tissue after RNA sequencing analysis; thus, 91 cases underwent immunohistochemical evaluation.

### 2.2. RNA Sequencing Analysis

Tissue samples were sectioned at a thickness of 10 μm from FFPE blocks and mounted on glass slides for RNA extraction and analysis by Macrogen (Seoul, Republic of Korea). Raw data generated from RNA sequencing were used for subsequent analyses. All RNA sequencing FASTQ files have been deposited under BioProject (https://www.ncbi.nlm.nih.gov/sra/PRJNA1206544, accessed on 27 March 2025) in the National Center for Biotechnology Information database (https://www.ncbi.nlm.nih.gov/). The datasets are available on GEO under accession number GSE293443. The quality of all FASTQ files was assessed using FastQC tools (version 0.12.1). Low-quality reads and adapter sequences were removed using Trimmomatic (version 0.39). The quality of all paired FASTQ files was verified using MultiQC (version 1.26). Before raw reads were aligned to the reference genome, a genome index was generated using GRCh38 and the annotation file GRCh38.113 (https://www.ensembl.org). Reads were aligned to the genome index using STAR (version 2.7.11b), and the resulting BAM files were sorted using SAMtools (version 1.21). Genes quantification and read counting were performed using the featureCounts function from the Subread package (version 2.0.8) in Bash; gene annotation was based on GRCh38.113. The generated count metrics were used for differentially expressed gene analysis with the DESeq2 package (version 1.46.0) in R software (4.4.2). Principal component analysis (PCA) plots were generated using the ggbiplot package in R. Correlation heatmaps were constructed with the pheatmap package, and volcano and box plots were produced using the ggplot2 package. Gene set enrichment analysis (GSEA) was conducted using Hallmark gene sets v2024, with 1000 permutations and no collapse, in GSEA (version 4.3.3) software (Broad Institute, Cambridge, MA, USA). GSEA results were considered statistically significant if both the *p*-value and false discovery rate (FDR) were less than 0.05. The Wilcoxon signed-rank test was used to compare paired groups using the Wilcoxon test function in R software.

### 2.3. TCGA Data Processing

For comparative analysis between the study cohort and publicly available transcriptomic data, mRNA expression profiles and clinical data were obtained from The Cancer Genome Atlas (TCGA) through cBioPortal for Cancer Genomics (https://www.cbioportal.org/) [12,13]. The dataset included progression-free and overall survival information for 109 laryngeal cancer samples. The dataset was imported into R software (version 2024.4.1.748) using the readxl package. RNA-seq data were filtered to match these patients, and gene identifiers were cleaned. KRT1 expression was extracted, transposed, and merged with clinical data. Patients were categorized by disease-free status, and KRT1 expression differences between recurrent and non-recurrent cases were analyzed using the Wilcoxon rank-sum test and visualized with ggplot2. Missing survival data were imputed using the median, and patients were stratified into high- and low-KRT1 expression groups based on median expression levels. Overall survival (OS) and progression-free survival (PFS) were analyzed using Kaplan–Meier survival curves. OS was defined as the time from diagnosis to death or last follow-up, while PFS was defined as the time from diagnosis to disease recurrence or last follow-up. Survival curves were generated using the survival and survminer R packages, with statistical significance assessed using the log-rank test.

### 2.4. Immunohistochemical Analysis

FFPE sections were prepared at a thickness of 3 μm from the blocks previously used for RNA sequencing. Immunohistochemical staining was performed using the automated Bond–Max system (Leica Microsystems, Bannockburn, IL, USA). Tissue pretreatment involved incubation with Bond Epitope Retrieval Solution 1 (citrate, pH 6.0) for 15 min, followed by incubation with a KRT1-specific antibody (1:200 dilution, ab185628; Abcam, Cambridge, UK). Negative controls were processed identically but without primary antibodies. Immunoreactivity was graded based on staining intensity as follows: negative (0), weak (1), moderate (2), or strong (3). The presence of characteristic dot-like KRT1 positivity was recorded separately from diffuse cytoplasmic staining. Two independent pathologists (L.K.H. and K.J.Y.) evaluated the immunohistochemical staining without knowledge of clinical records. In cases of disagreement, a collaborative review was conducted to reach a consensus.

### 2.5. Cell Culture and Transfection

The SNU 1041 cell line was purchased from the Korean Cell Line Bank (Seoul, Republic of Korea). The PCl1 cell line [14] was provided by Dr. MW Sung (Myung-Whun Sung, Seoul National University, Seoul, Republic of Korea). All HNSCC cell lines were maintained in RPMI 1640 medium supplemented with 10% fetal bovine serum (FBS), 100 U/mL penicillin, and 100 µg/mL streptomycin in 100 mm × 20 mm culture dishes (Corning Inc., Corning, NY, USA). For KRT1 knockdown in HNSCC cells, small interfering RNAs (siRNAs) were used. Cells were seeded in six-well plates at a density of 2.0 × 10^5^ cells per well and transfected with either a KRT1-specific siRNA (Bioneer Corporation, Daejeon, Republic of Korea) or a negative control siRNA (Qiagen, Germantown, MD, USA) using Lipofectamine (Invitrogen, Thermo Fisher Scientific, Waltham, MA, USA) for 48 h at 37 °C. The KRI1-specific siRNA sequences were as follows: Sense, 5′-CGA ACG UGA GUG UGU CUG U-3′; Antisense, 5′-ACA GAC ACA CUC ACG UUC G-3′.

### 2.6. RNA Isolation and Reverse-Transcription Polymerase Chain Reaction (RT-PCR)

Total RNA was extracted from SNU-1041 and PCl1 HNSCC cells using TRIzol reagent (Invitrogen), in accordance with the manufacturer’s protocol. Reverse transcription was performed using 1 µg of total RNA, M-MLV reverse transcriptase (Invitrogen), 1 µL of 2 mM dNTP mix (Enzynomics Co., Ltd., Daejeon, Republic of Korea), 2 µL of 0.1 M dithiothreitol (Invitrogen), 4 µL of 5× first-strand buffer (Invitrogen), 1 µL of RNase inhibitor (Promega Corporation), and 1 µL of oligo(dT) primer (Bioneer Corporation). cDNA was amplified using primers specific for KRT1 and glyceraldehyde-3-phosphate dehydrogenase (GAPDH) (Bioneer Corporation). PCR was conducted with GoTaq DNA Polymerase and 5× Green GoTaq reaction buffer (Promega Corporation). The primer sequences were as follows: KRT1 forward, 5′-CCC TCC TGG TGG CAT ACA AG-3′; KRT1 reverse, 5′-GTT GGT CCA CTC TCC TTC GG-3′; GAPDH forward, 5′-ACC ACA GTC CAT GCC ATC AC-3′; and GAPDH reverse, 5′-TCC ACC CTG TTG CTG TA-3′. PCR products were separated by electrophoresis on a 1% agarose gel containing ethidium bromide.

### 2.7. Protein Isolation and Western Blot Analysis

Cells were lysed using radioimmunoprecipitation assay buffer (Biosesang Inc., Sungnam, Republic of Korea). Protein concentrations were determined using the bicinchoninic acid assay. Protein lysates (20–30 µg) were separated by sodium dodecyl sulfate-polyacrylamide gel electrophoresis on 10–12% gels and electrophoretically transferred to polyvinylidene fluoride membranes. Membranes were incubated for 1 h at room temperature with 5% bovine serum albumin (Bioshop Canada Inc., Burlington, ON, Canada) in Tris-buffered saline with Tween 20 (TBS-T); this was followed by four washes (15 min each) using TBS-T. Specific proteins were detected using primary antibodies against β-actin (cat. no. 3700, Cell Signaling Technology, Inc., Danvers, MA, USA), KRT1 (cat. no. ab185628; Abcam, Cambridge, UK), cleaved caspase-3 (cat. no. 9664), cleaved caspase-7 (cat. no. 9491), and poly (ADP-ribose) polymerase (PARP; cat. no. 5625; Cell Signaling Technology, Inc., Danvers, MA, USA), and X-linked inhibitor of apoptosis protein (XIAP; cat. no. sc-11426; Santa Cruz Biotechnology, Dallas, TX, USA). Additional antibodies included those targeting SLUG (SNAI2; cat. no. 9585; Cell Signaling Technology, Inc., Danvers, MA, USA), vimentin (cat. no. ab898; Abcam, Cambridge, UK), ZEB1 (cat. no. A301-922A; Bethyl Laboratories, Inc. Montgomery, TX, USA), ZEB2 (cat. no. AV33693; Sigma-Aldrich, St. Louis, MO, USA), CD44 (cat. no. ab189524; Abcam, Cambridge, UK), and CD133 (cat. no. ab1998; Abcam, Cambridge, UK). Each primary antibody was diluted 1:1,000 and incubated with membranes at 4 °C for 24 h. Horseradish peroxidase (HRP)-conjugated secondary antibodies, either anti-rabbit (cat. no. 7074; Cell Signaling Technology, Inc.) or anti-mouse (cat. no. 7076, Cell Signaling Technology, Inc.), were diluted 1:2,000 and incubated with membranes at room temperature for 1 h.

Immunoreactive proteins were visualized using an enhanced chemiluminescence detection system for HRP (EMD Millipore, Billerica, MA, USA). Image analysis was performed using the LAS 4000 luminescent image analyzer (Fujifilm, Tokyo, Japan).

### 2.8. Cell Invasion Assay

Viable cells transfected with KRT1-specific siRNA or a negative control siRNA were seeded at a density of 2.0 × 10^5^ cells in 120 µL of a 0.2% bovine serum albumin suspension in the upper chamber. The lower chamber was filled with 400 µL of 0.2% bovine serum albumin containing fibronectin (cat. no. 361635; Calbiochem, San Diego, CA, USA) as a chemoattractant. After 24 h of incubation, the cells that had migrated to the bottom surface of the Transwell membrane were stained using Diff–Quik solution (Sysmex, Kobe, Japan). Cell invasion ability was assessed by quantifying the number of cells that passed through an 8.0 µm pore Transwell invasion apparatus (Costar, Cambridge, UK). Cells were counted in five randomly selected microscope fields. Results are presented as the mean ± standard error of the number of invading cells per field from three independent experiments.

### 2.9. Cell Migration (Wound Healing) Assay

Cells were seeded in each well of a Culture-Insert (Ibidi GmbH) at a density of 1.5 × 10^5^ cells per well 24 h after transfection with KRT1-specific siRNA or a negative control siRNA. After incubation for 12 h, inserts were removed; cell migration was assessed by capturing images at 0, 4, 8, and 12 h using an inverted microscope. Migration distances were normalized to 1 cm, based on three randomly selected sites.

### 2.10. Apoptosis Assay

Apoptosis was assessed using an allophycocyanin (APC) annexin V assay. Cells transfected with a KRT1-specific siRNA or a negative control siRNA were collected 48 h after transfection using trypsin. Cells were washed twice with phosphate-buffered saline and resuspended in binding buffer (cat. no. 556454; BD Biosciences, San Jose, CA, USA). After the addition of APC annexin V (cat. no. 550474) and 7-amino-actinomycin D (7-AAD; cat. no. 559925; BD Biosciences, San Jose, CA, USA), cells were incubated for 15 min in the dark and resuspended in 400 µL of binding buffer. Flow cytometry analysis was performed using a FACS Calibur flow cytometer (BD Biosciences) and BD CellQuest version 3.3 software (Becton Dickinson). Data analysis was conducted using WinMDI version 2.9 (The Scripps Research Institute, San Diego, CA, USA).

### 2.11. Cell Irradiation and Cisplatin Treatment

For irradiation, cells were exposed to γ-irradiation at a dose of 10 Gy (137 Cs, 2.875 Gy/min) using a Gammacell 3000 Elan (Therathronics, Kanata, ON, Canada) at room temperature, followed by incubation at 37 °C. For cisplatin treatment, cells were incubated with cisplatin (0.5 mg/mL; Dong-A, Seoul, Republic of Korea) at a concentration of 10 µg/mL for 24 h at 37 °C.

### 2.12. Statistical Analysis

Student’s *t*-test was used to assess statistical significance in experimental comparisons. Survival curves were generated using the Kaplan–Meier method and Cox proportional hazards modeling; they were compared using the log-rank test. Statistical analyses were conducted using Statistical Package for the Social Sciences (SPSS) version 29.0 (IBM, Armonk, NY, USA). *p*-values less than 0.05 were considered statistically significant.

## 3. Results

### 3.1. KRT1 Expression Analysis Through RNA Sequencing and TCGA Dataset

**Figure 1:**
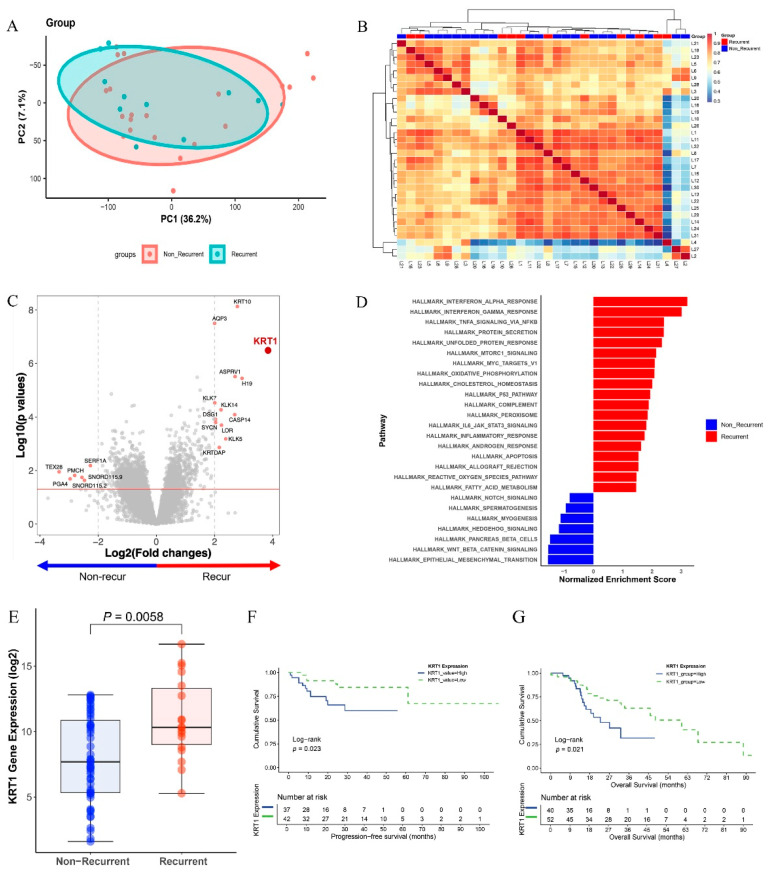
Differentially expressed genes (DEGs) in recurrence and non-recurrence groups: (**A**) Principal component analysis (PCA) plot comparing recurrence and non-recurrence groups. (**B**) Heatmap showing unsupervised hierarchical clustering of 11 recurrent and 21 non-recurrent cases based on gene expression profiles. (**C**) Volcano plot displaying DEGs based on recurrence, with fold changes and *p*-values. (**D**) Gene set enrichment analysis (GSEA) demonstrating enrichment of the interferon-alpha response and epithelial–mesenchymal transition pathways from KEGG gene sets in recurrence and non-recurrence groups, respectively. (**E**) Box plots showing elevated KRT1 expression levels in the recurrence group. (**F**) Kaplan–Meier estimates of progression-free survival according to KRT1 expression levels using TCGA datasets. (**G**) Kaplan–Meier estimates of overall survival according to KRT1 expression levels using TCGA datasets.

RNA sequencing analysis was performed on FFPE tissue sections from recurrent and non-recurrent specimens to identify differentially expressed genes associated with recurrence. Principal component analysis revealed partial overlap between the recurrence and non-recurrence groups (Figure 1A). Similarly, unsupervised hierarchical clustering of normalized gene expression values demonstrated comparable patterns without distinct separation between groups (Figure 1B).

Among the differentially expressed genes, KRT1 exhibited the highest differential expression between the two groups (*p* < 0.001, Figure 1C). Gene set enrichment analysis indicated that the interferon-alpha response pathway was most strongly upregulated in the recurrence group, whereas the EMT signaling pathway was significantly upregulated in the non-recurrence group (Figure 1D). The difference in KRT1 expression between the recurrence and non-recurrence groups was statistically significant at the transcriptome level in our patient cohort (*p* = 0.0058, Figure 1E).

Survival analysis using the TCGA dataset indicated that high KRT1 expression was associated with shorter progression-free survival (PFS), with statistical significance (*p* < 0.05, Figure 1F). Additionally, overall survival (OS) analysis demonstrated significantly worse outcomes in the high KRT1 expression group (*p* < 0.05, Figure 1G). The median follow-up for the entire cohort was 31.7 months for PFS and 33.7 months for OS. For the groups stratified by Keratin-1 expression, the median follow-up for PFS, the median follow-up was 24.4 months in the KRT1-high group and 36.0 months in the KRT1-low group. Similarly, OS was 22.5 months in the KRT1-high group and 44.6 months in the KRT1-low group.

### 3.2. Clinical Significance of KRT1 Expression in HNSCC Tissues

**Figure 2:**
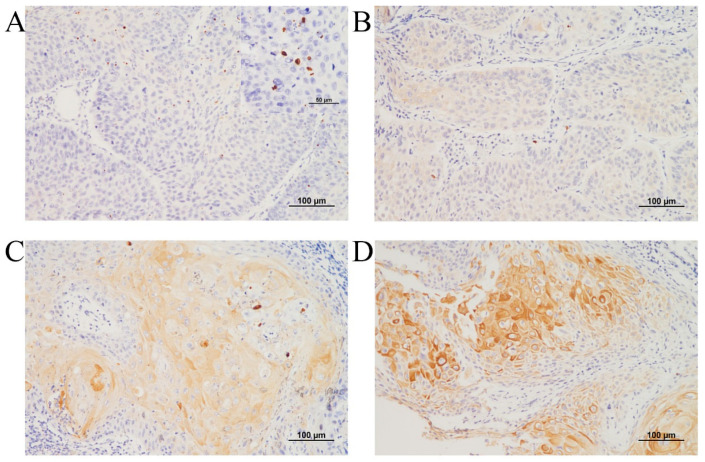
Representative microscopy images of KRT1 expression in laryngeal squamous cell carcinoma: (**A**) Negative KRT1 expression with dot-like positivity, shown in the inset box. (**B**) KRT1 expression of intensity 1. (**C**) KRT1 expression of intensity 2. (**D**) KRT1 expression of intensity 3. (**A**–**D**: immunohistochemistry, original magnification ×200).

After KRT1 had been identified via RNA sequencing and TCGA dataset analysis, immunohistochemical evaluation was performed on tissue samples from patients diagnosed with advanced laryngeal cancer who underwent definitive CCRT. The study population predominantly consisted of male patients (89 out of 91) with a mean age of 66.7 years (range: 41.3–85 years). KRT1 expression was assessed using a three-tier intensity grading system and by evaluating the presence of characteristic dot-like positivity (Figure 2A–D).

**Figure 3:**
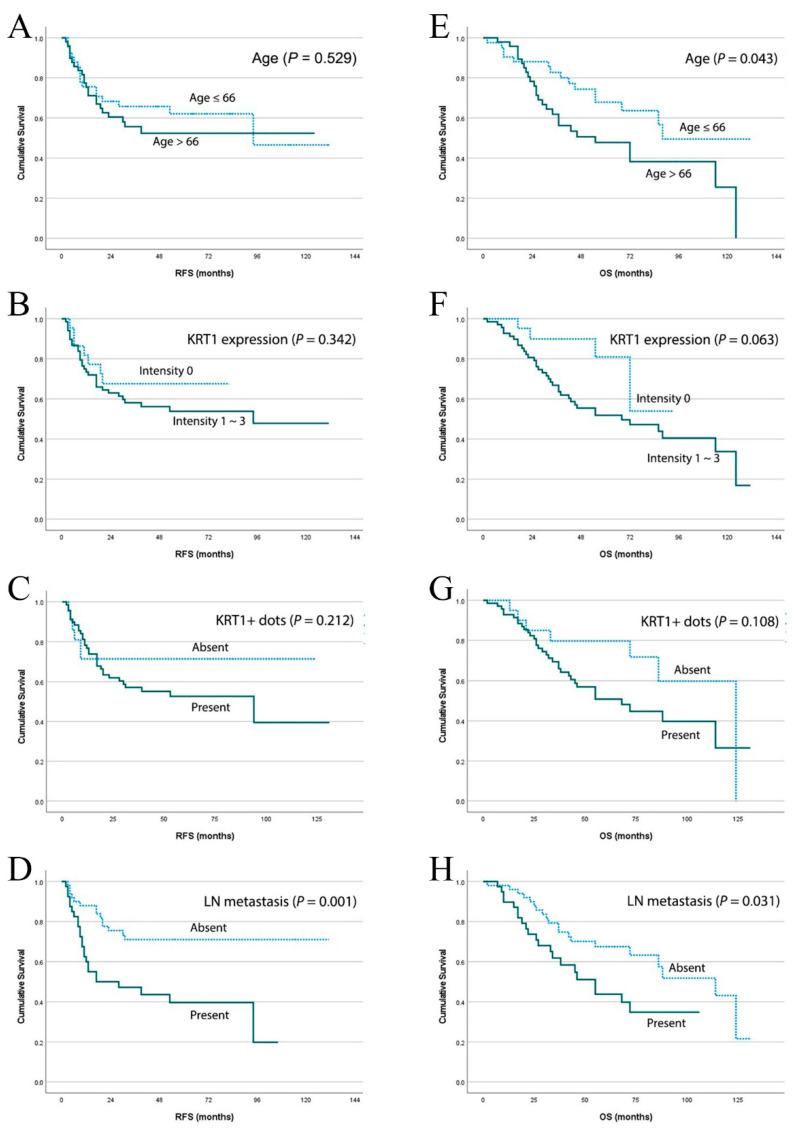
Survival analysis based on clinicopathological parameters in advanced laryngeal cancers. Kaplan–Meier estimates of recurrence-free survival according to (**A)** age, (**B**) positive KRT1 expression, (**C**) presence of KRT1-positive dots, and (**D**) lymph node metastasis. Kaplan–Meier estimates of overall survival according to (**E**) age, (**F**) positive KRT1 expression, (**G**) presence of KRT1-positive dots, and (**H**) lymph node metastasis in the same patient cohort.

Kaplan–Meier analysis demonstrated that lymph node metastasis was significantly associated with shorter recurrence-free survival and overall survival (*p* = 0.001 and *p* = 0.031, respectively; Figure 3). Advanced age, positive KRT1 expression, and the presence of KRT1-positive dots exhibited trends toward shorter recurrence-free survival periods, although these associations did not reach statistical significance. In overall survival analysis, advanced age significantly predicted worse survival outcomes (*p* = 0.043), whereas positive KRT1 expression showed a marginally significant predictive effect (*p* = 0.063). Consistent with the recurrence-free survival findings, the presence of KRT1-positive dots demonstrated a tendency toward worse overall survival.

### 3.3. KRT1 Knockdown in Human HNCCC Cell Lines

**Figure 4:**
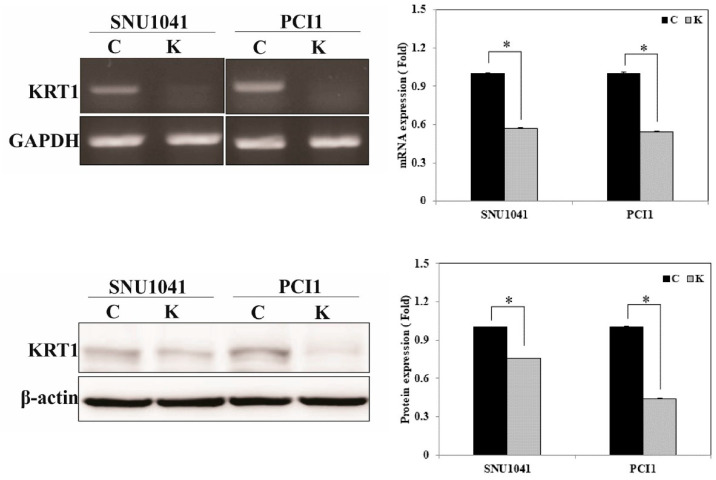
KRT1 inhibition in human head and neck cancer cells. mRNA and protein levels were reduced in KRT1-knockdown SNU-1041 and PCl1 cells compared with negative control siRNA-transfected cells, as demonstrated by RT-PCR (**upper panel**) and Western blotting (**lower panel**). RT-PCR, reverse transcription-polymerase chain reaction; siRNA, small interfering RNA; C, negative control siRNA-transfected cells; K, KRT1-specific siRNA-transfected cells; * *p* < 0.05.

To investigate the role of KRT1 in tumor progression in vitro, KRT1-specific siRNA was used to inhibit the endogenous KRT1 expression in the SNU-1041 and PCl1 human HNSCC cell lines. mRNA and protein levels of KRT1 were lower in cells transfected with KRT1-specific siRNA than in those transfected with negative control siRNA (Figure 4, Supplementary Material for the uncropped whole Western blot images).

### 3.4. KRT1 Knockdown Suppresses Tumor Cell Invasion, Migration, and EMT-Related Genes in Human HNCCC Cell Lines

**Figure 5:**
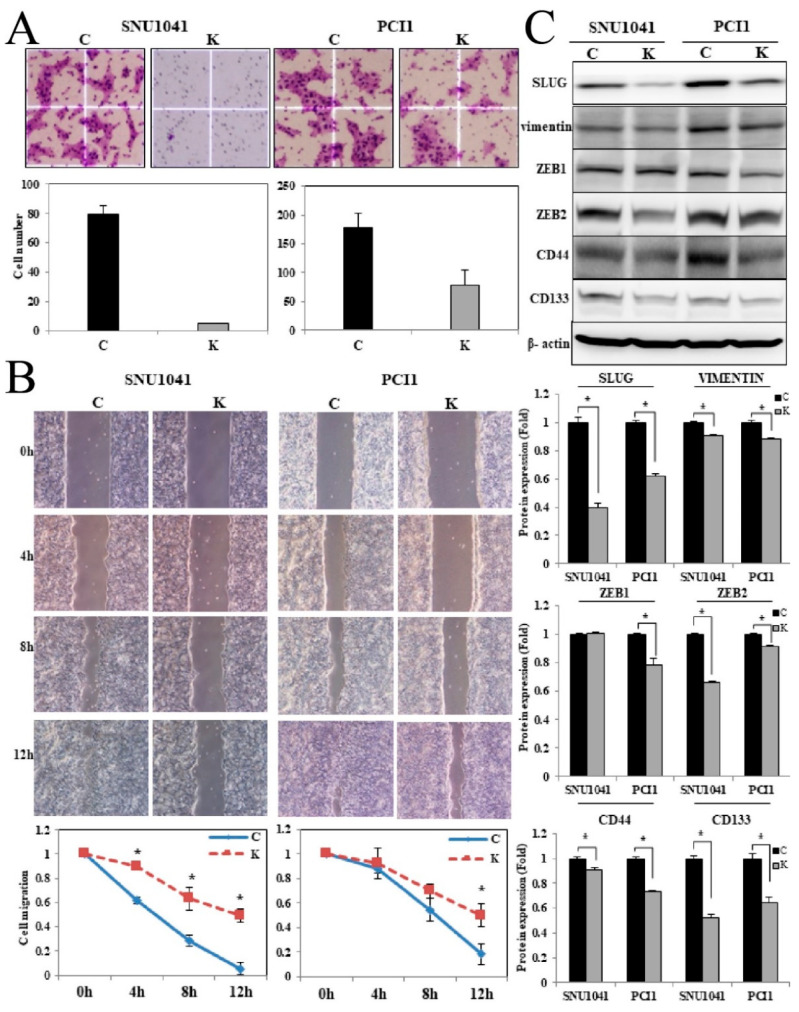
Effects of KRT1 knockdown on cell invasion, migration, and epithelial–mesenchymal transition-related transcription factors in human head and neck cancer cells: (**A**) In the cell invasion assay, KRT1-knockdown SNU-1041 and PCl1 cells exhibited reduced invasion compared with negative control cells. Stained invading cells were quantified (bar graph; mean ± standard error of three independent experiments). (**B)** Cell migration was lower in KRT1-knockdown SNU-1041 and PCl1 cells than in negative control cells. Relative healing distances were measured to assess migration at three random sites. (**C**) KRT1 knockdown reduced the expression levels of SLUG, vimentin, ZEB2, and CD44 in both SNU-1041 and PCl1 cells. ZEB1 expression showed a slight decrease, whereas CD133 expression remained unchanged. C, negative control siRNA-transfected cells; K, KRT1-specific siRNA-transfected cells. SLUG, Snail family transcriptional repressor 2; ZEB1, zinc finger E-box-binding homeobox 1; ZEB2, zinc finger E-box-binding homeobox 2.; * *p* < 0.05.

In the cell invasion assay, KRT1-knockdown SNU-1041 cells exhibited a significant reduction in invasion compared with negative control SNU-1041 cells (Figure 5A; 1 vs. 79.25 ± 5.8, *p* < 0.05). Similarly, KRT1-knockdown PCl1 cells showed significantly reduced invasion (79 ± 25.2) compared with negative control PCl1 cells (177.8 ± 25.6, *p* < 0.05) (Figure 5A).

Cell migration was assessed by measuring artificial wound closure over time. The wound gap significantly narrowed in negative control SNU-1041 and PCl1 cells at 4, 8, and 12 h, whereas KRT1-knockdown cells retained an open wound gap at 12 h (Figure 5B, *p* < 0.05).

To investigate potential mechanisms underlying the effect of KRT1 knockdown, the expression levels of EMT-related proteins, including Snail family transcriptional repressor 2 (SLUG), vimentin, and zinc finger E-box-binding homeobox 1 and 2 (ZEB1 and ZEB2), were evaluated. Additionally, the expression levels of CD44 and CD133 were analyzed. SLUG, vimentin, ZEB2, and CD44 levels were reduced in KRT1-knockdown cells, whereas ZEB1 showed a slight decrease, and CD133 expression remained unchanged in both SNU-1041 and PCl1 cells (Figure 5C, Supplementary Material for the uncropped whole Western blot images).

### 3.5. KRT1 Knockdown Enhances Tumor Cell Apoptosis in Human HNCCC Cell Lines

**Figure 6:**
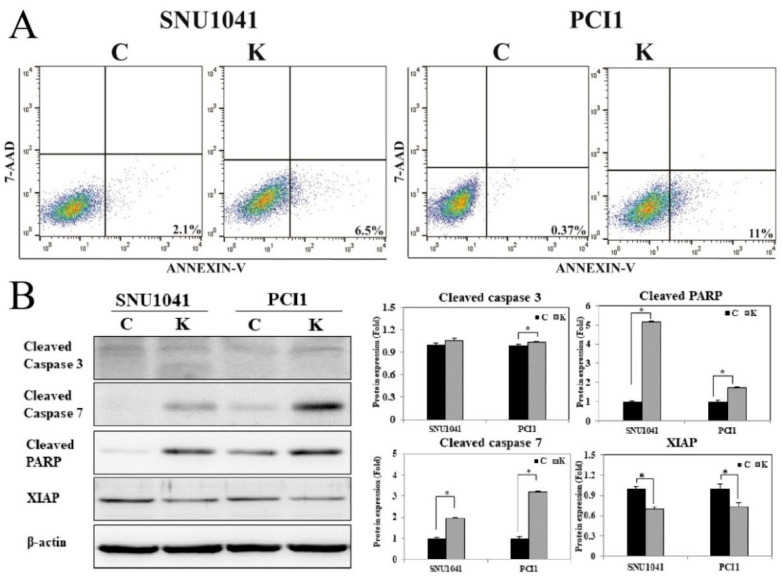
Effects of KRT1 knockdown on cell apoptosis in human head and neck cancer cells: (**A**) In the cell apoptosis assay, representative dot plots demonstrated that KRT1-knockdown SNU-1041 and PCl1 cells exhibited higher levels of apoptosis compared with control cells. (**B**) Levels of cleaved caspase-3, caspase-7, and cleaved PARP were elevated in KRT1-knockdown SNU-1041 and PCl1 cells compared with control cells. XIAP expression was lower in KRT1-knockdown SNU-1041 and PCl1 cells than in control cells. C, negative control siRNA-transfected cells; K, KRT1-specific siRNA-transfected cells; PARP, poly(ADP-ribose) polymerase; XIAP, X-linked inhibitor of apoptosis protein; 7-AAD, 7-amino-actinomycin D; * *p* < 0.05.

An annexin V apoptosis assay was performed to evaluate the effects of KRT1 knockdown on apoptosis. Flow cytometry analysis demonstrated a significantly increased proportion of apoptotic cells in KRT1-knockdown SNU-1041 and PCl1 cells compared with controls (Figure 6). The proportion of early apoptotic cells induced by transfection with KRT1-specific siRNA was higher than that observed in negative control siRNA-transfected cells (6.5% vs. 2.1% and 11% vs. 0.37% in SNU-1041 and PCl1 cells, respectively). To confirm the effects of KRT1 knockdown on tumor cell apoptosis, apoptosis-regulatory protein levels were analyzed after siRNA treatment. Levels of cleaved caspase-3, caspase-7, and PARP were elevated in KRT1-knockdown SNU-1041 and PCl1 cells compared with negative controls (Figure 6B, Supplementary Material for the uncropped whole Western blot images). Additionally, levels of XIAP were reduced in KRT1-knockdown cells compared with negative controls (Figure 6B). These findings indicate that KRT1 knockdown enhances tumor cell apoptosis by modulating apoptosis-regulatory proteins such as caspase-3, caspase-7, and PARP, as well as inhibitory apoptosis regulators such as XIAP, in human HNSCC cells.

### 3.6. KRT1 Knockdown Enhances Radiosensitivity and Chemosensitivity and in Human HNCCC Cell Lines

**Figure 7:**
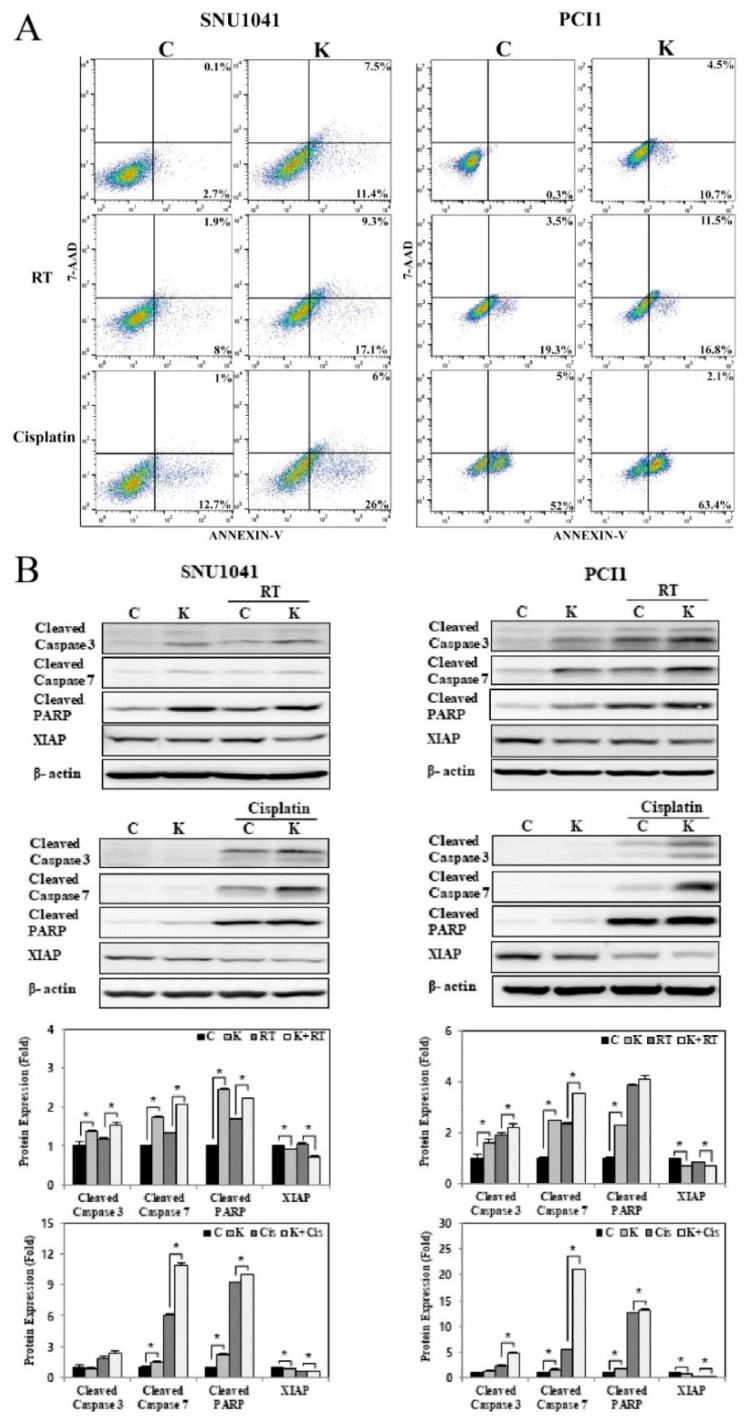
Effects of KRT1 knockdown on radiosensitivity and chemosensitivity in human head and neck cancer cells: (**A**) The combination of KRT1 knockdown with either 10 Gy radiation or 10 ug/mL cisplatin resulted in increased apoptosis in SNU-1041 and PCl1 cells relative to control cells treated with 10 Gy radiation or 10 ug/mL cisplatin alone. (**B**) KRT1-knockdown cells exhibited higher levels of cleaved caspase-3, caspase-7, and cleaved PARP expression than control cells after treatment with 10 Gy radiation or 10 ug/mL cisplatin. C, negative control siRNA-transfected cells; K, KRT1-specific siRNA-transfected cells; Cis, cisplatin; siRNA, small interfering RNA; RT, radiation; PARP, poly(ADP-ribose) polymerase; XIAP, X-linked inhibitor of apoptosis protein; 7-AAD, 7-amino-actinomycin D; * *p* < 0.05.

To assess the effects of KRT1 knockdown on radiosensitivity, KRT1-specific siRNA or negative control siRNA was transfected into SNU-1041 and PCl1 cells. After 48 h, cells were irradiated at a dose of 10 Gy. The combination of KRT1 knockdown and radiation resulted in significantly higher apoptosis than radiation alone in both SNU-1041 and PCl1 cells (Figure 7A). The proportions of early and late apoptotic cells in KRT1-knockdown cells irradiated at 10 Gy were higher than those observed in negative control irradiated cells at 10 Gy (26.4% vs. 9.9% and 28.3% vs. 22.8% in SNU-1041 and PCl1 cells, respectively). Expression levels of cleaved caspase-3, caspase-7, and PARP after radiation treatment were higher in KRT1-knockdown cells than in control cells (Figure 7B, Supplementary Material for the uncropped whole Western blot images). XIAP expression was reduced in KRT1-knockdown cells compared with controls (Figure 7B). These findings suggest that the combination of KRT1 knockdown and radiotherapy exerts synergistic apoptotic effects in human HNSCC cells.

To explore the effects of KRT1 knockdown on chemosensitivity through apoptosis induction, SNU-1041 and PCl1 cells transfected with KRT1-specific siRNA or negative control siRNA were treated with cisplatin (10 µg/mL) at 48 h post-transfection. The combination of KRT1 knockdown and cisplatin resulted in significantly higher apoptosis than chemotherapy alone in both SNU-1041 and PCl1 cells (Figure 7A). Expression levels of cleaved caspase-3, caspase-7, and PARP after cisplatin treatment were elevated in KRT1-knockdown cells compared with controls (Figure 7B). XIAP expression was significantly reduced in KRT1-knockdown cells relative to control cells (Figure 7B). These findings indicate that KRT1 knockdown enhances the apoptotic effects of chemotherapy in human HNSCC cells.

## 4. Discussion

NCCN guidelines recommend either surgery or chemoradiation for advanced laryngeal cancer, such a Stage III or IV, as standard treatment options. The treatment of advanced laryngeal cancer substantially impacts patient quality of life, particularly in terms of voice and swallowing function. Therefore, treatment strategies to preserve organ function are essential. The combination of chemotherapy and radiation has enabled a large proportion of patients with advanced laryngeal cancer to retain organ function [1,2]. However, debate persists regarding the optimal treatment approach for advanced laryngeal cancer since survival rates have not been improved [2,4,15,16]. According to data from the National Cancer Database, the 5-year overall survival rate for laryngeal cancer declined from 68.1% to 64.7% between 1985 and 2001 [17]. Development of chemoradiation has improved patient morbidity in terms of organ preservation; however, a study has shown that overall and disease-specific survival rates after surgery, such as total laryngectomy, were better [18]. This trend suggests that chemoradioresistance is a primary factor contributing to decreased survival over time. The identification of novel therapeutic targets associated with chemoradioresistance in laryngeal cancer is crucial to not only improving patient outcomes but also aiding first-line treatment decision-making between extensive surgery and chemoradiation.

To identify genes associated with laryngeal cancer recurrence, RNA sequencing was performed in the present study, leading to the identification of KRT1. Few studies have examined KRT1 in cancer; its expression has been reported in premalignant and malignant oral lesions [7,8,19]. Another study demonstrated that KRT80 is involved in esophageal carcinogenesis and contributes to chemoresistance [20]. Some studies have shown that KRT1 is correlated with chemotherapy resistance and sensitivity in human ovarian cancer cell lines and nasopharyngeal cancer [9,10]. In this study, KRT1 expression was significantly higher in the recurrence group, according to single-center data from 33 specimens. The in-house cohort group was homogenous, which excluded the patients who underwent primary surgery. Analysis of the larger TCGA dataset further confirmed that KRT1 expression was significantly elevated in the recurrence group. Survival analysis using TCGA data demonstrated relationships of KRT1 expression with recurrence and survival. Among the 91 patients analyzed for clinicopathological characteristics, lymph node metastasis was the only factor significantly associated with recurrence and survival. Although the T stage is generally considered a key factor in recurrence, all T1-stage cases were excluded from this study, potentially introducing bias. Survival analysis using in-house data indicated that KRT1 expression was associated with worse survival, but statistical significance was marginal. Additionally, in-house data suggested that KRT1-positive dots were associated with a tendency toward recurrence and poor survival. The inclusion criteria for this study were limited to patients who underwent CCRT, suggesting that increased KRT1 expression is associated with chemoradioresistance.

After the investigation of KRT1 function in recurrence and survival using in vivo datasets, in vitro experiments were conducted for further validation. The results indicated that KRT1 enhances cell invasion and migration in HNSCC cells. EMT is a process in which epithelial cells acquire a motile mesenchymal phenotype. As expected, KRT1 knockdown inhibited EMT. To increase invasive and migratory capacity, primary tumor cells lose adhesion molecule expression, resulting in metastasis and poor prognosis [21,22]. According to a previous study, ZEB1 and ZEB2 repress E-cadherin expression, whereas ZEB2 directly interacts with the vimentin promoter in human epithelial breast tumor cells [23]. Given that KRT1 knockdown inhibited both vimentin and ZEB2 expression, the differential expression of ZEB1 and ZEB2 should have been further examined in this study.

The in vitro apoptosis assay demonstrated that KRT1 knockdown increased apoptosis in HNSCC cells. The combination of radiation or chemotherapy with KRT1 knockdown appeared to have a synergistic effect, leading to elevated levels of apoptosis-regulatory proteins such as caspase-3, caspase-7, and PARP. However, XIAP, an apoptosis inhibitor, was reduced after KRT1 knockdown. Notably, XIAP expression significantly decreased when KRT1 knockdown was combined with radiation or cisplatin treatment. A previous study indicated that XIAP may predict response to cisplatin-based chemotherapy [24]. These findings suggest that KRT1 plays a role in cisplatin chemoresistance and radioresistance.

The primary limitation of this study was the relatively small sample size of the in-house cohort. However, the results were supported by a larger dataset from the TCGA dataset, which does not discrete groups according to treatment method, but still confirmed the associations of KRT1 expression with recurrence and survival. Further validation in larger treatment-homogeneous clinical cohorts is required to establish the clinical significance of KRT1 as a predictive biomarker of CRT. Despite this limitation, this study is the first to identify KRT1 as a potential biomarker in advanced laryngeal cancer using strict inclusion criteria. The study focused on patients with Stage III or IV disease who underwent definitive CCRT to specifically investigate chemoradioresistance and recurrence. In vitro experiments further confirmed the relationship between KRT1 expression and chemoradioresistance. The potential mechanisms by which KRT1 may contribute to chemoradioresistance in HNSCC, including its interaction with known factors, represent an important direction for future research. Although additional studies are necessary, KRT1 shows promise as a predictive biomarker for patient selection before chemoradiation as the first treatment. Furthermore, investigations of KRT1 and its downstream signaling pathways may improve treatment outcomes in terms of laryngeal preservation and oncological survival.

## 5. Conclusions

This study provides compelling evidence that KRT1 plays a key role in laryngeal cancer recurrence and chemoradioresistance. Therefore, KRT1 may serve as a biomarker for predicting recurrence and guiding patient selection for either chemoradiation or surgery. Furthermore, with additional investigations of its molecular mechanisms and clinical applicability, KRT1 inhibition could represent a promising therapeutic strategy, enhancing both laryngeal function preservation and survival outcomes.

## Data Availability

The datasets generated and analyzed during the current study, available on the NCBI repository (https://www.ncbi.nlm.nih.gov/sra/PRJNA1206544, accessed on 27 March 2025), are available on GEO under accession number GSE293443.

## Supplementary Materials

The following supporting information can be downloaded at: https://www.mdpi.com/article/10.3390/curroncol32090520/s1, File S1: Uncropped whole Western blot images.

## Abbreviations

The following abbreviations are used in this manuscript:

KRT1	Keratin-1
PCR	polymerase chain reaction
OS	overall survival
RFS	recurrence-free survival
EMT	Epithelial–mesenchymal transition
HNSCC	head and neck squamous cell carcinoma
CCRT	concurrent chemoradiotherapy
FFPE	formalin-fixed paraffin-embedded
TCGA	The Cancer Genome Atlas
PFS	progression-free survival
APC	allophycocyanin
7-AAD	7-amino-actinomycin D
SLUG	Snail family transcriptional repressor 2
ZEB	zinc finger E-box-binding homeobox
