# Supplementary material for: Expression of Keratin-1 Predicts Recurrence and Treatment Response in Advanced Laryngeal Cancer: A Potential Therapeutic Target
Source: Curr Oncol. 2025 Sep 17;32(9):520. doi: 10.3390/curroncol32090520 (PMC12468875; doi:10.3390/curroncol32090520)
Supplement: Supplementary file 1 [file curroncol-32-00520-s001.zip › curroncol-3818357-supplementary.pdf]

The uncropped original Western blot images showing all the bands with molecular weight markers, as shown in Figure 4, 5C, 6B, 7B. These images are unprocessed original gel images. However, during the Western blot, prior to applying the antibody, there were gels which we performed cutting based on the molecular weight range of the target protein, according to indicated data sheet provided by the antibody supplier. As a result, these images do not represent the full-length blot.

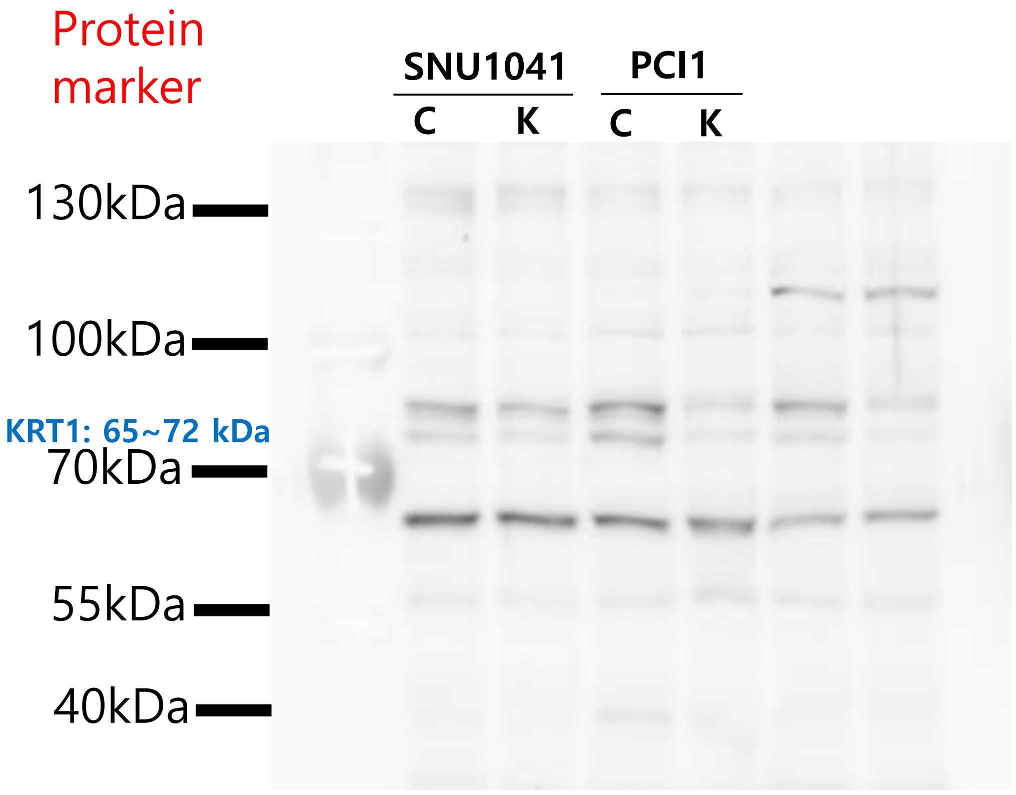

Uncropped whole western blot showing expression level of KRT 1 on SNU-1041 and PCl1 cells of figure 4 is showed with molecular weight markers.

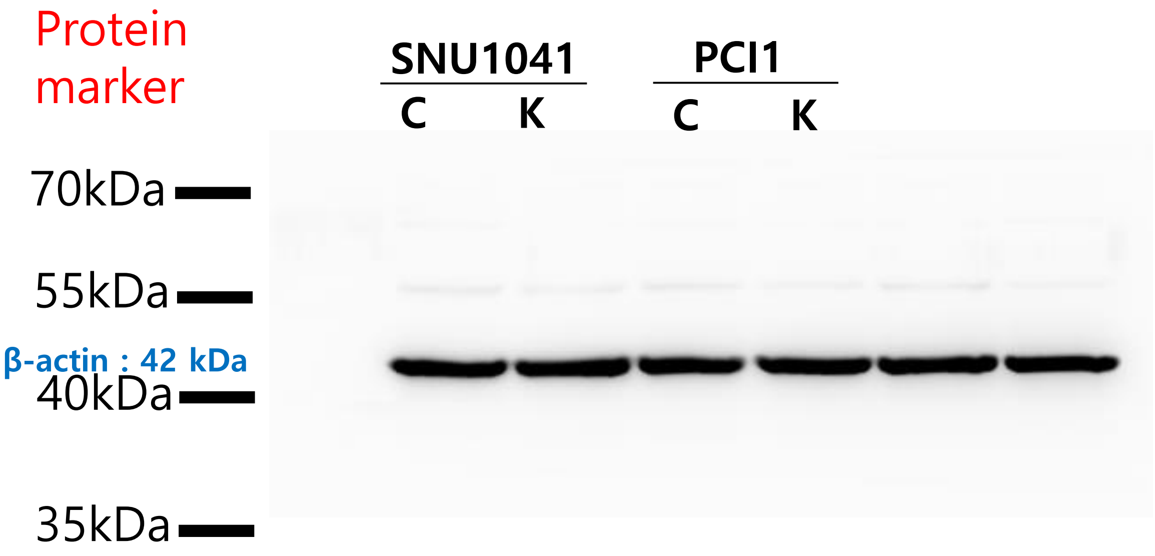

Uncropped whole western blot showing expression level of β-actin on SNU-1041 and PCl1 cells of figure 4 is showed with molecular weight markers.

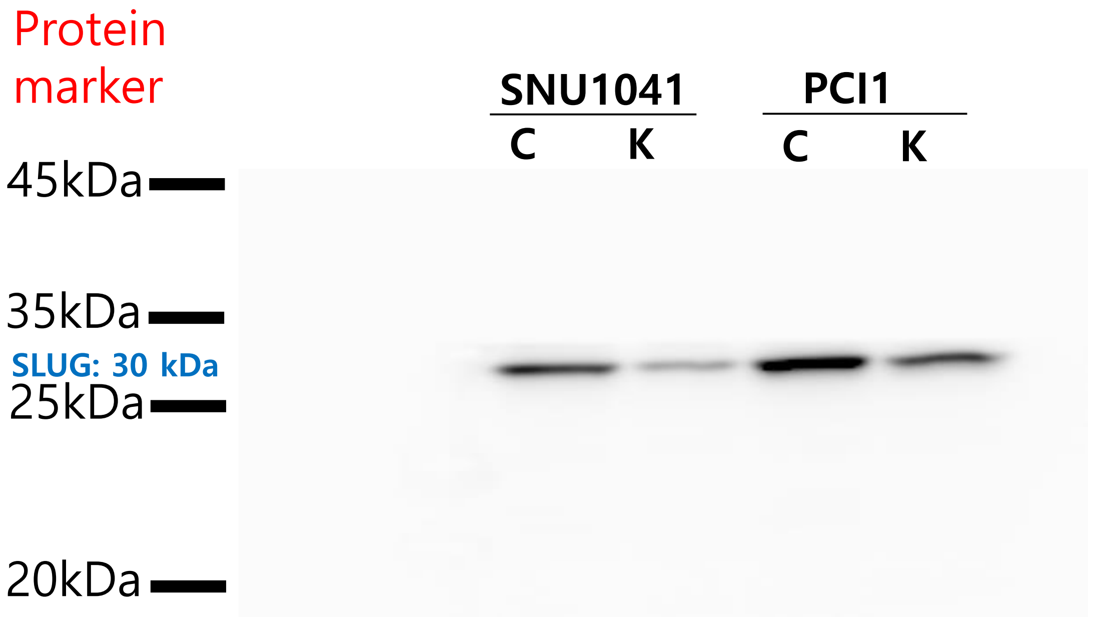

Uncropped whole western blot showing expression level of SLUG on SNU-1041 and PCl1 cells of figure 5 is showed with molecular weight markers.

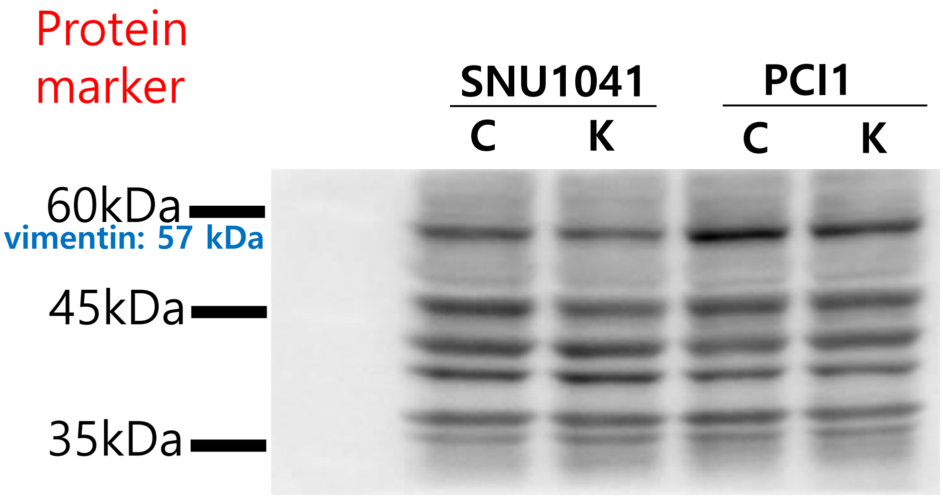

Uncropped whole western blot showing expression level of vimentin on SNU-1041 and PCl1 cells of figure 5 is showed with molecular weight markers.

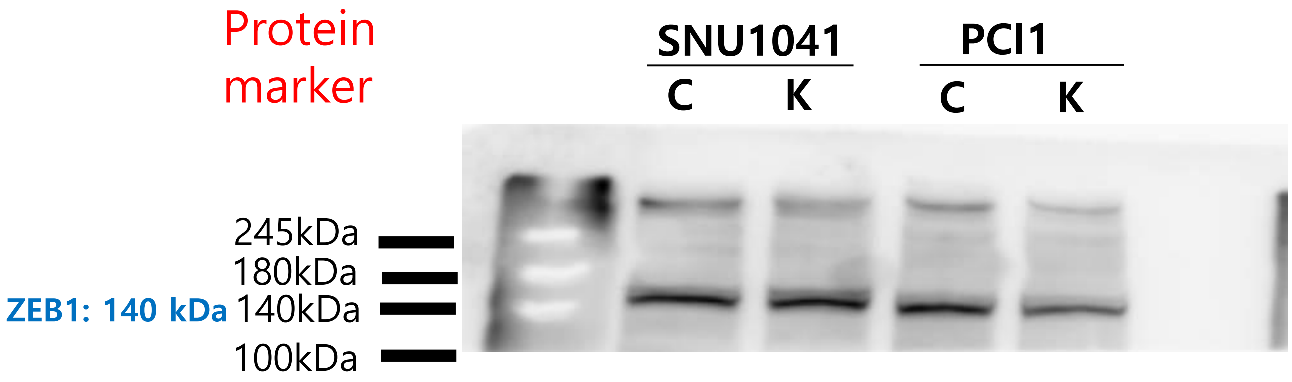

Uncropped whole western blot showing expression level of ZEB1 on SNU-1041 and PCl1 cells of figure 5 is showed with molecular weight markers.

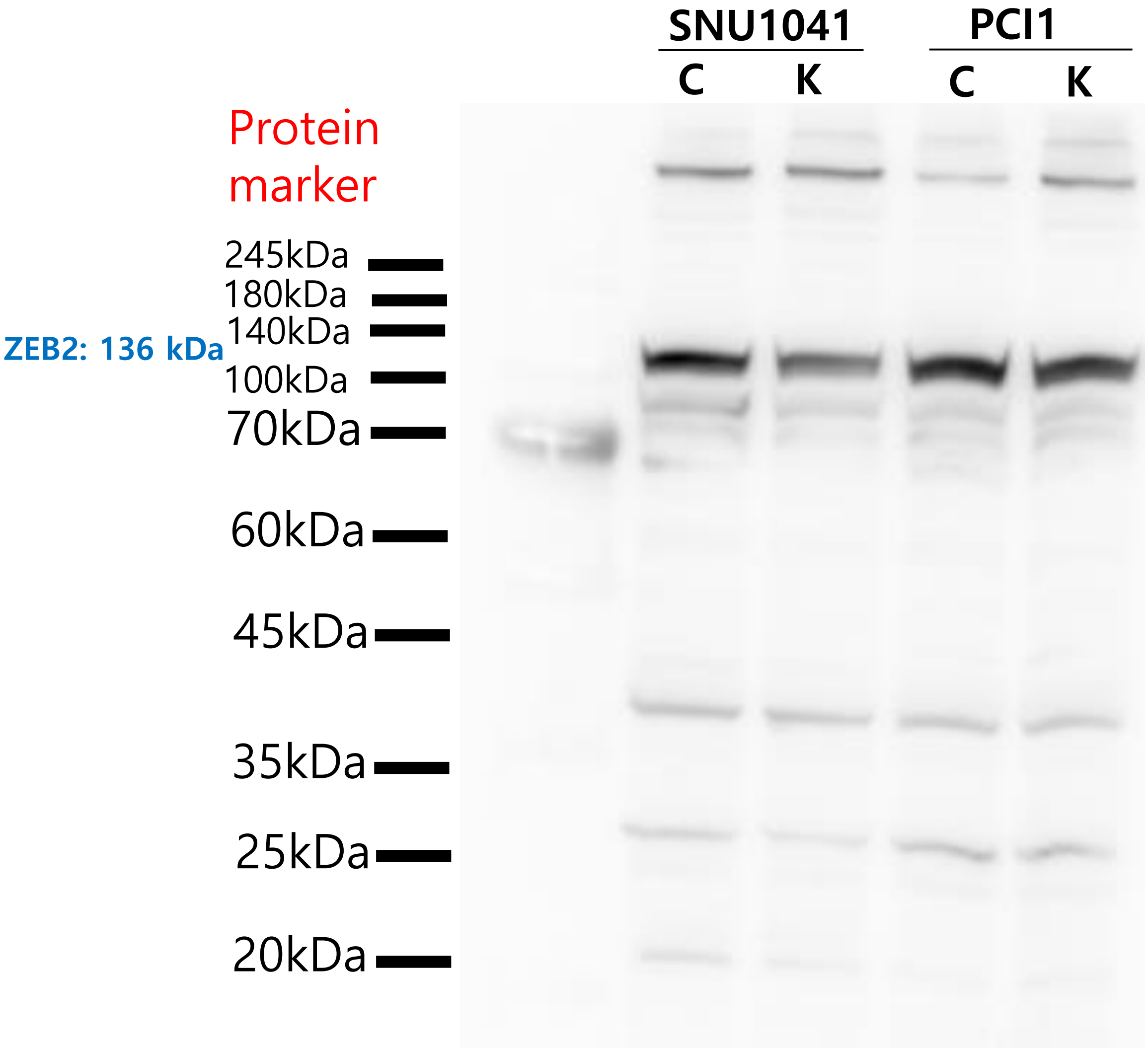

Uncropped whole western blot showing expression level of ZEB2 on SNU-1041 and PCl1 cells of figure 5 is showed with molecular weight markers.

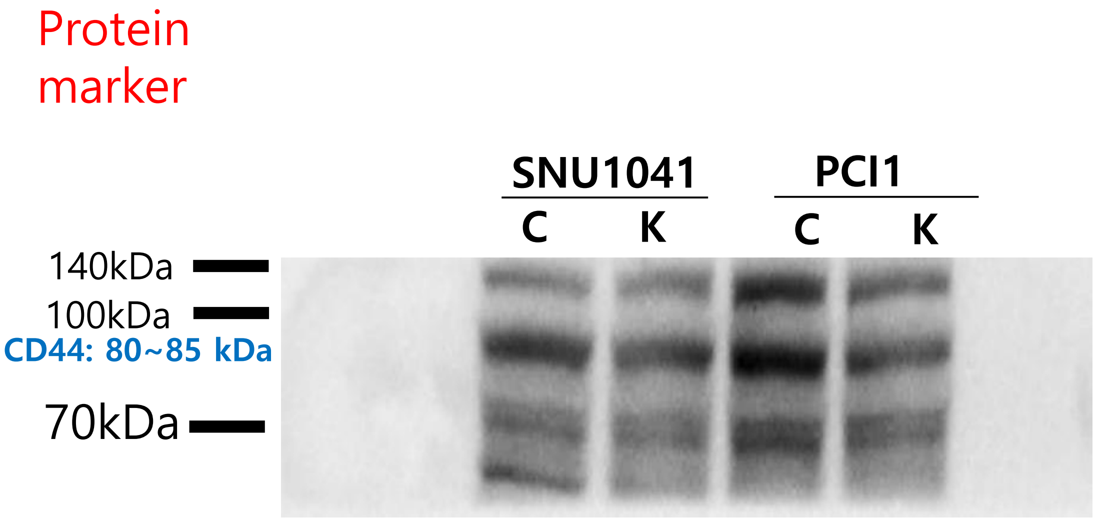

Uncropped whole western blot showing expression level of CD44 on SNU-1041 and PCl1 cells of figure 5 is showed with molecular weight markers.

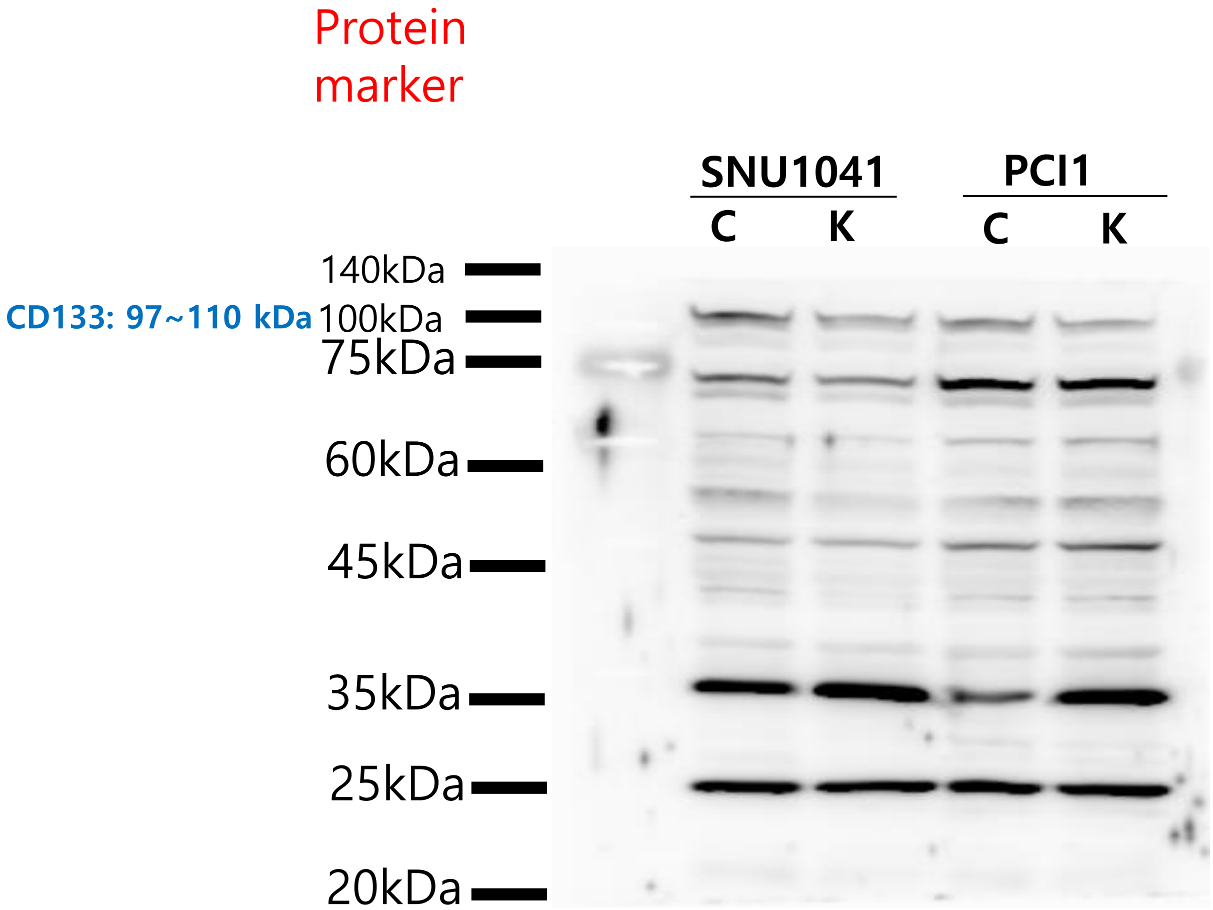

Uncropped whole western blot showing expression level of CD133 on SNU-1041 and PCl1 cells of figure 5 is showed with molecular weight markers.

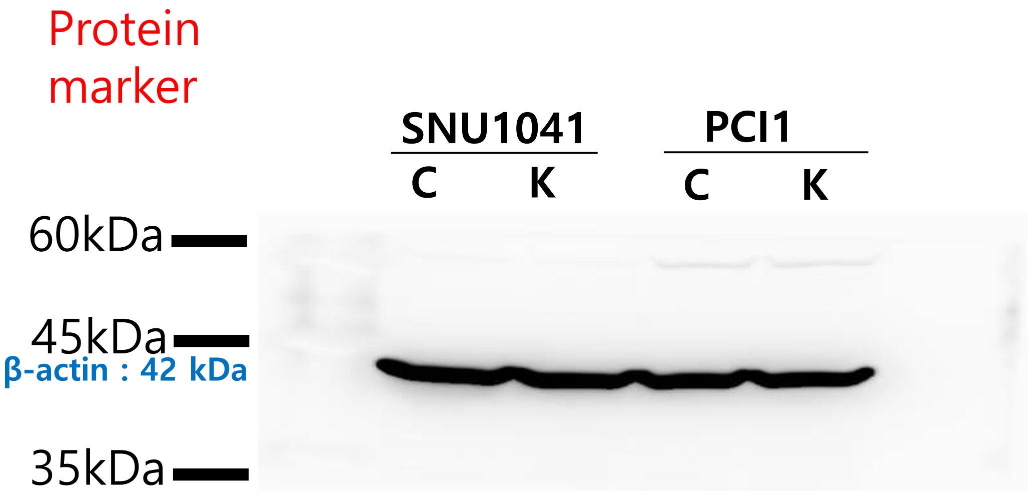

Uncropped whole western blot showing expression level of β-actin on SNU-1041 and PCl1 cells of figure 5 is showed with molecular weight markers.

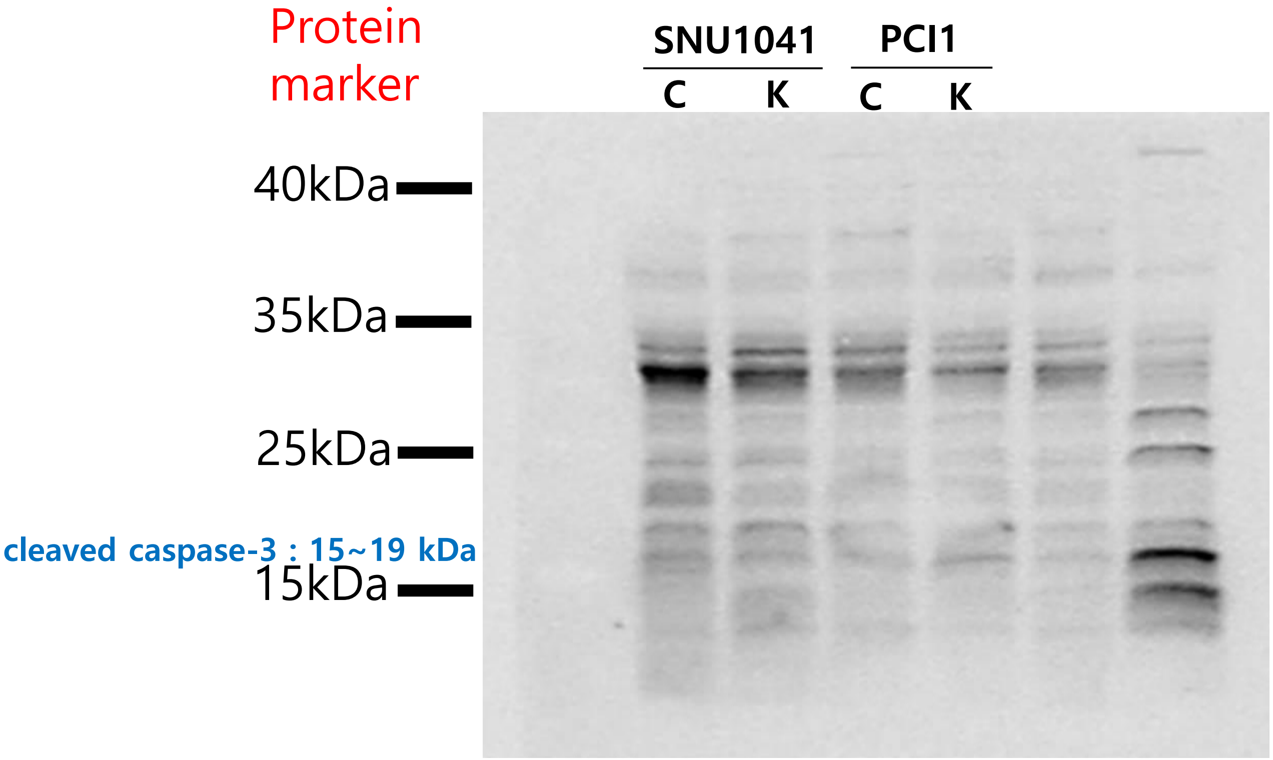

Uncropped whole western blot showing expression level of cleaved caspase-3 on SNU-1041 and PCl1 cells of figure 6 is showed with molecular weight markers.

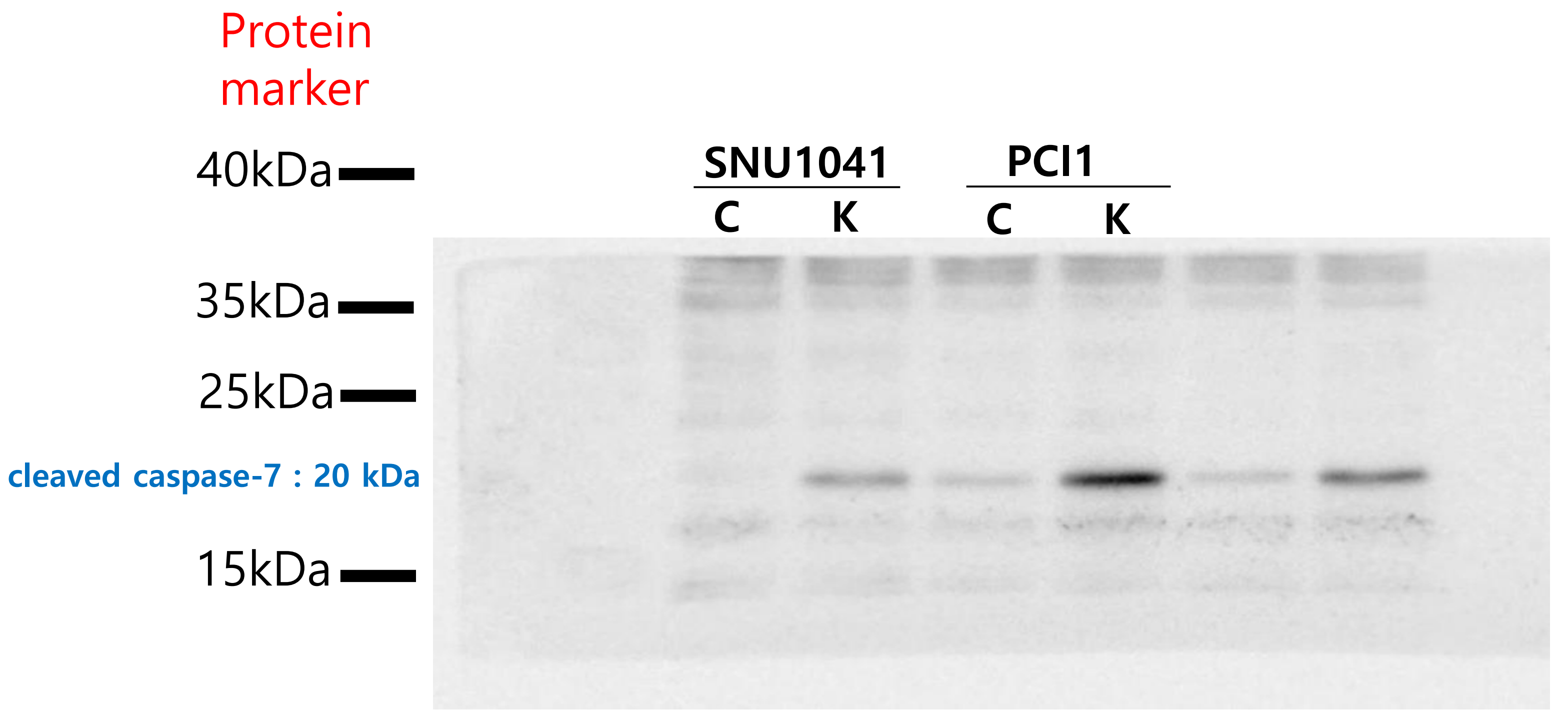

Uncropped whole western blot showing expression level of cleaved caspase-7 on SNU-1041 and PCl1 cells of figure 6 is showed with molecular weight markers.

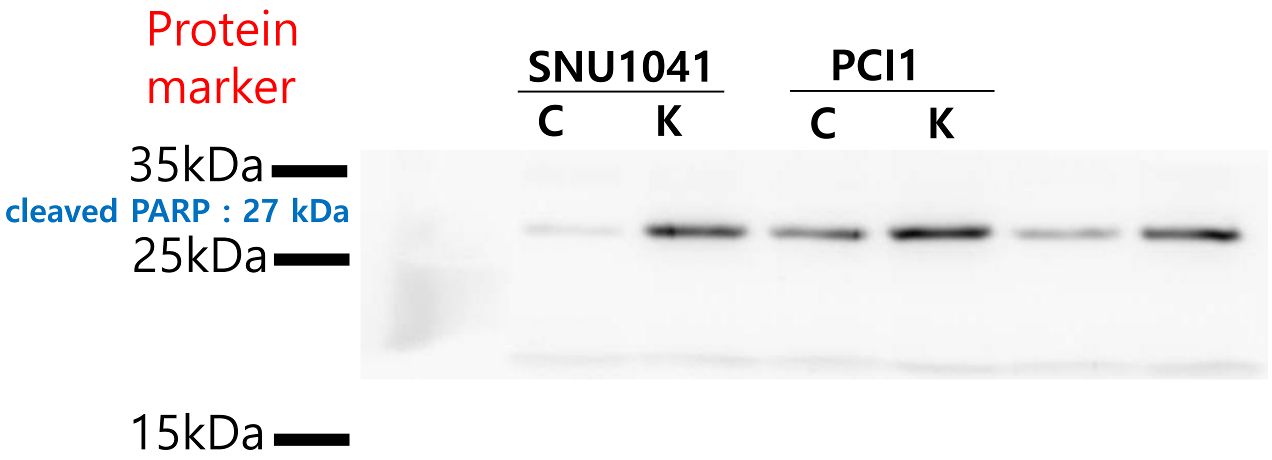

Uncropped whole western blot showing expression level of cleaved PARP on SNU-1041 and PCl1 cells of figure 6 is showed with molecular weight markers.

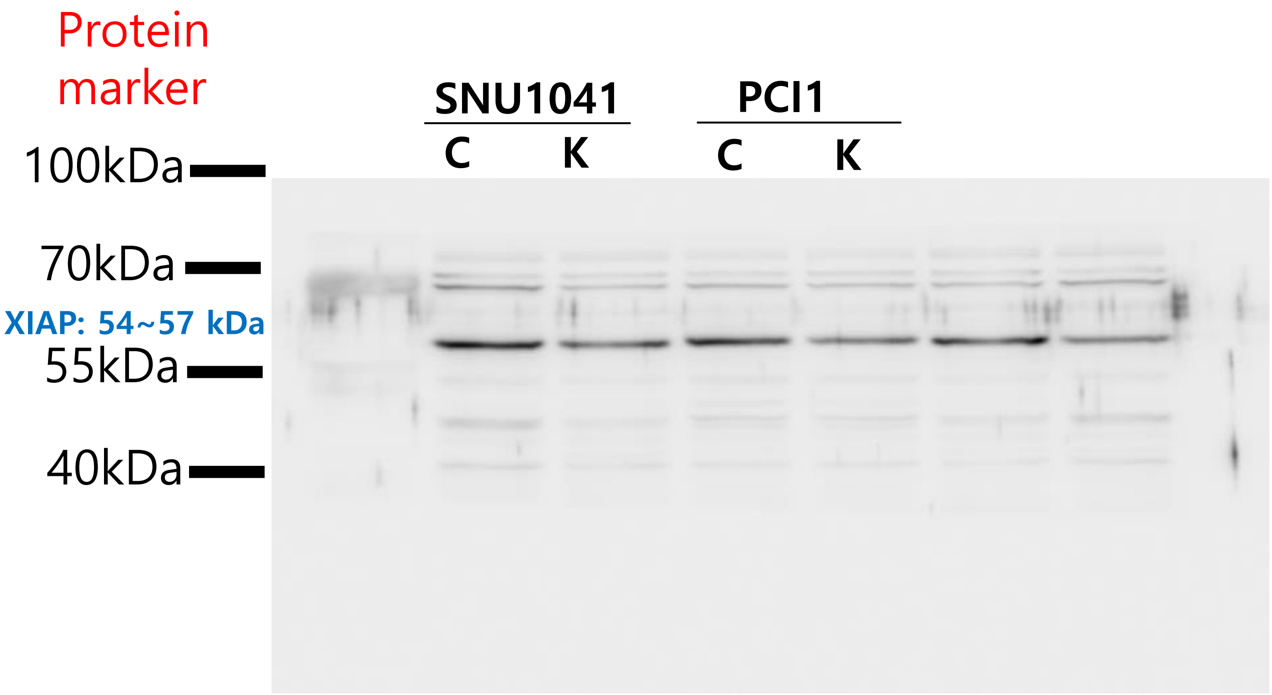

Uncropped whole western blot showing expression level of XIAP on SNU-1041 and PCl1 cells of figure 6 is showed with molecular weight markers.

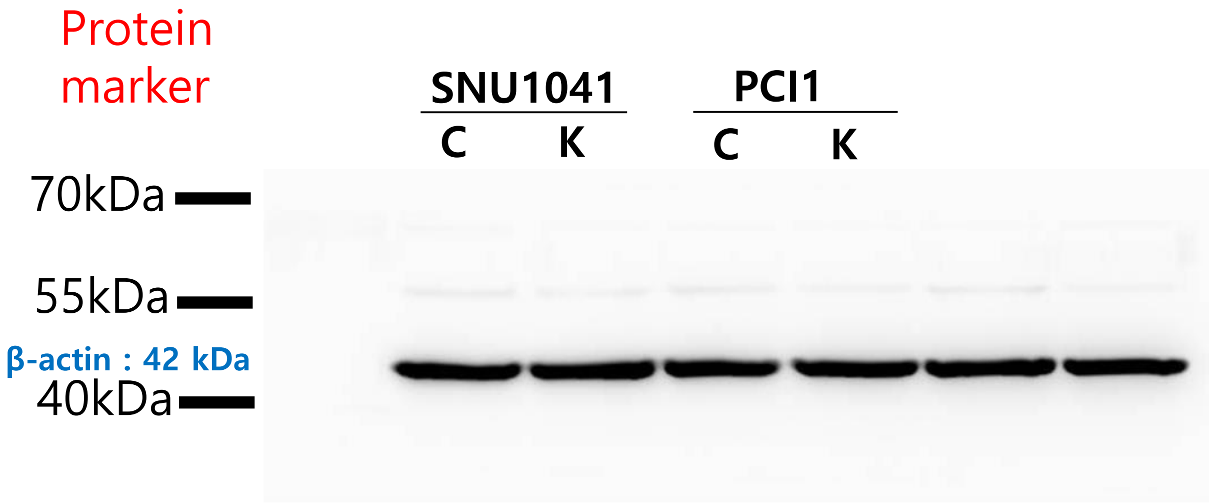

Uncropped whole western blot showing expression level of β-actin on SNU-1041 and PCl1 cells of figure 6 is showed with molecular weight markers.

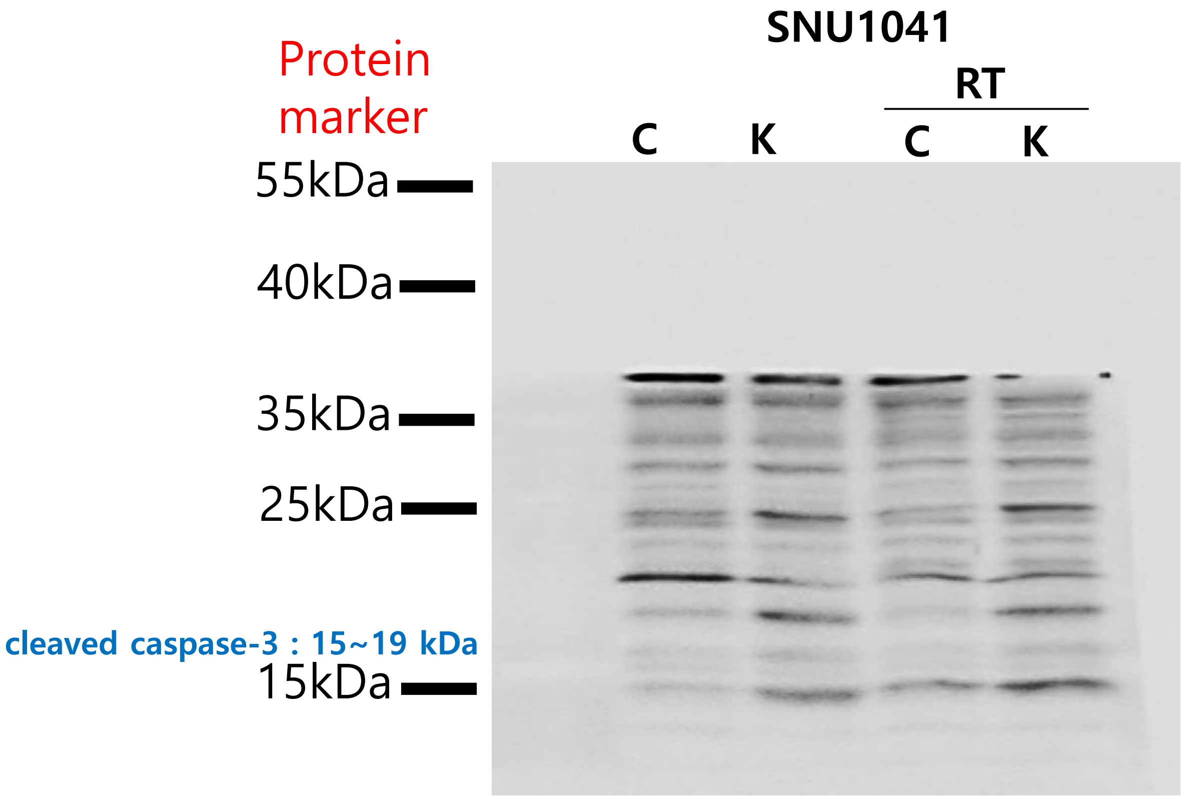

Uncropped whole western blot showing expression level of cleaved caspase-3 on SNU-1041 cells with RT of figure 7 is showed with molecular weight markers.

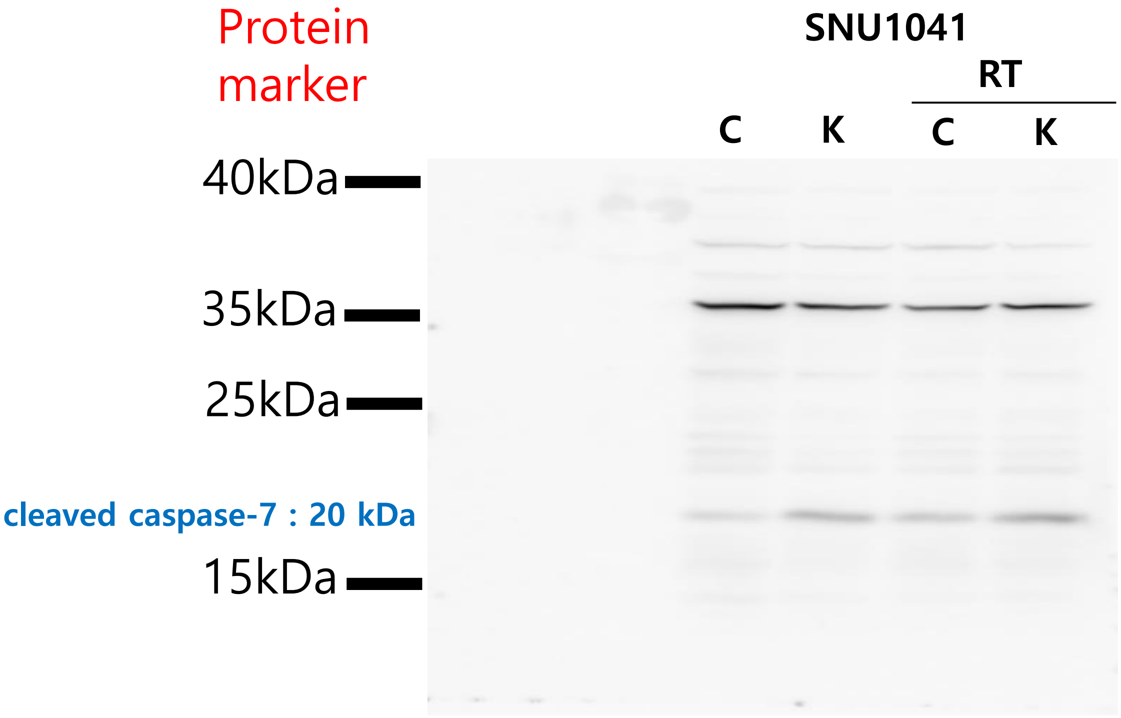

Uncropped whole western blot showing expression level of cleaved caspase-7 on SNU-1041 cells with RT of figure 7 is showed with molecular weight markers.

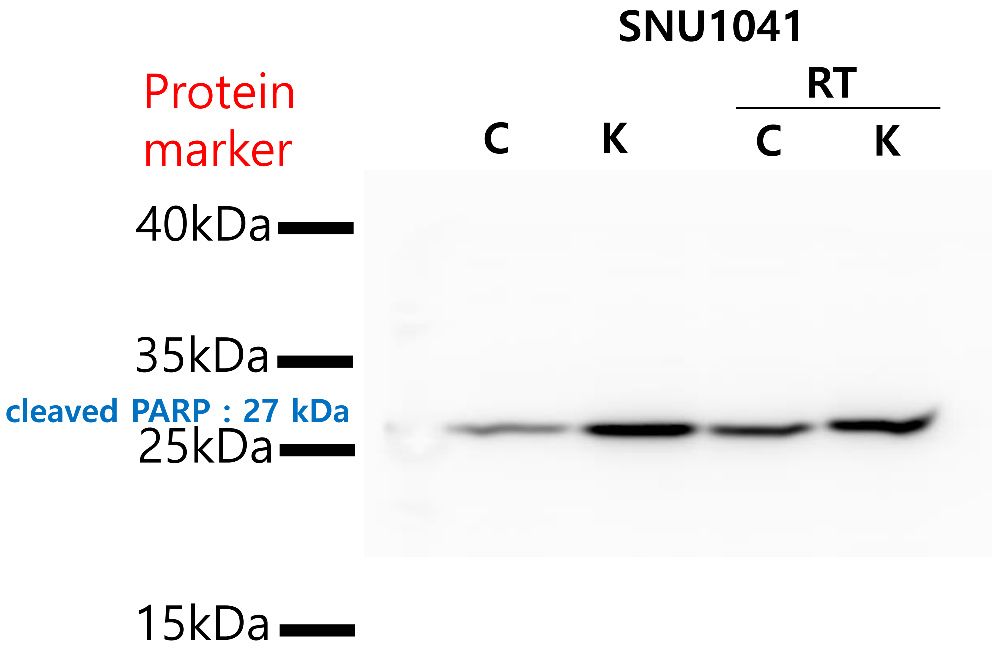

Uncropped whole western blot showing expression level of cleaved PARP on SNU-1041 cells with RT of figure 7 is showed with molecular weight markers.

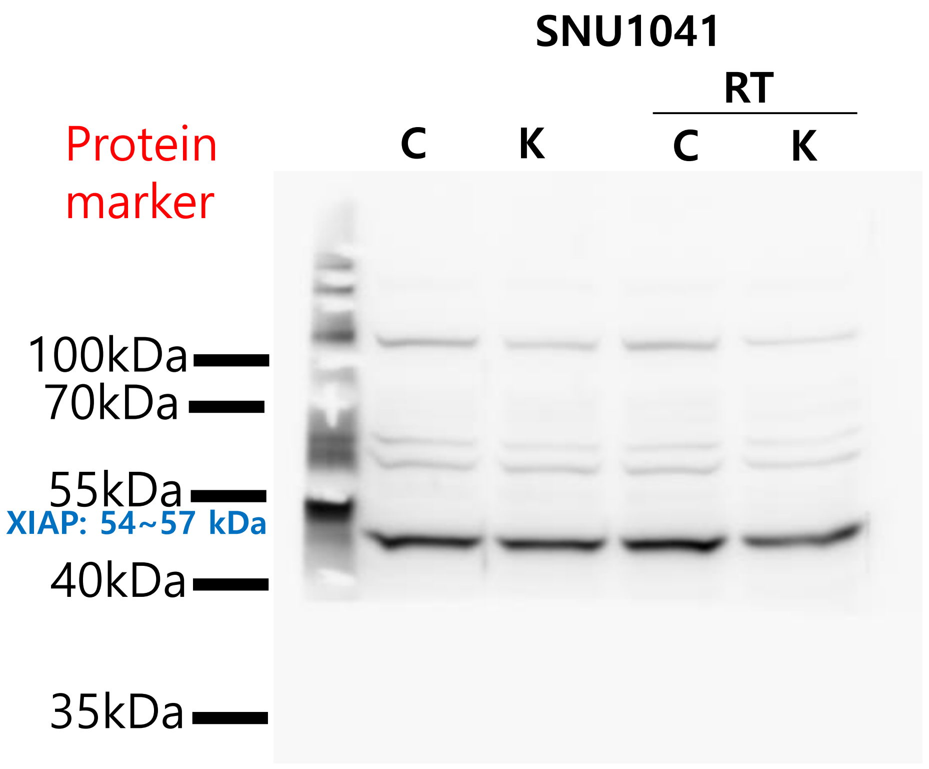

Uncropped whole western blot showing expression level of XIAP on SNU-1041 cells with RT of figure 7 is showed with molecular weight markers.

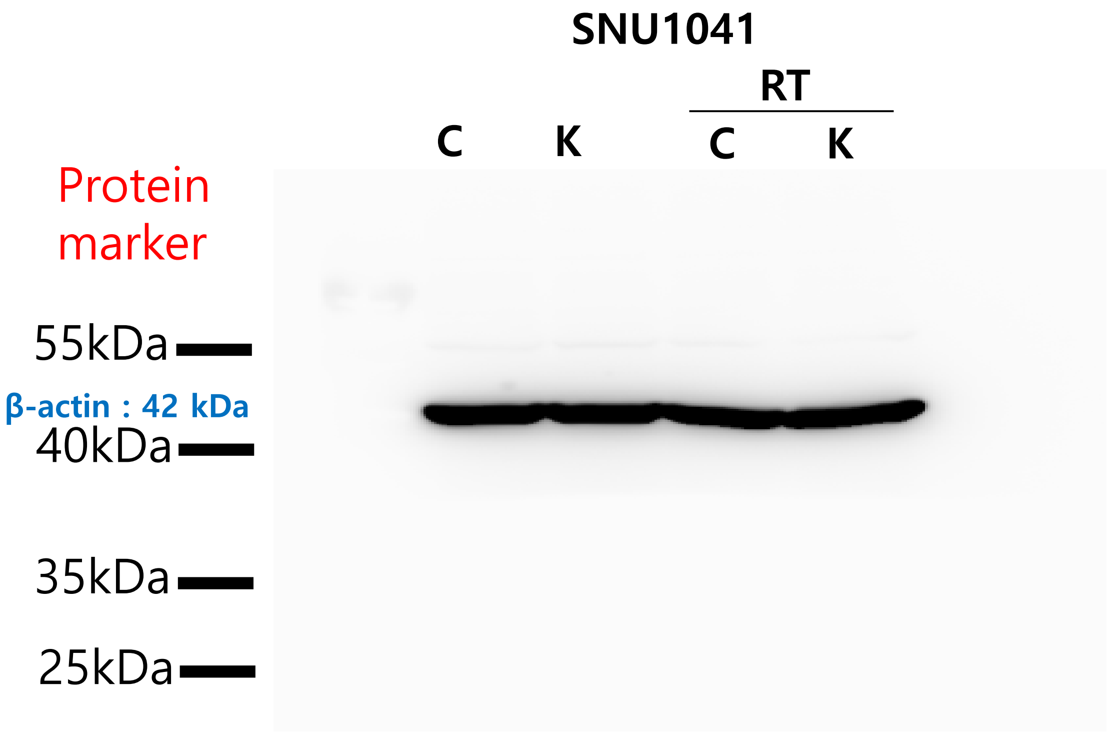

Uncropped whole western blot showing expression level of β-actin on SNU-1041 cells with RT of figure 7 is showed with molecular weight markers.

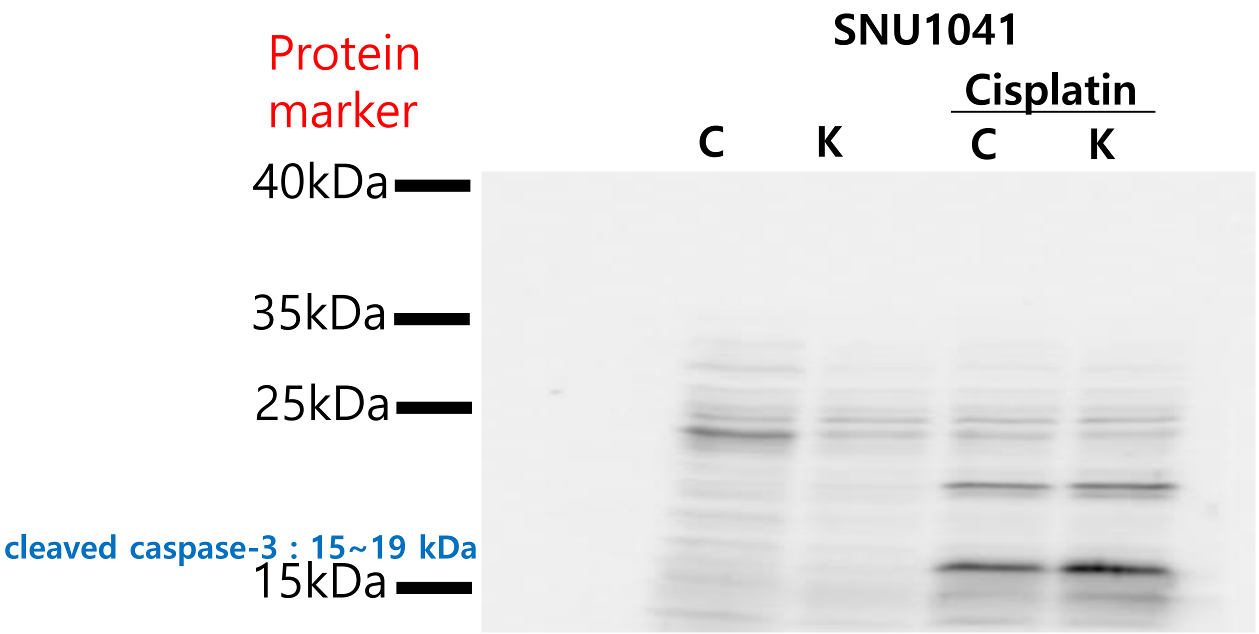

Uncropped whole western blot showing expression level of cleaved caspase-3 on SNU-1041 cells with cisplatin of figure 7 is showed with molecular weight markers.

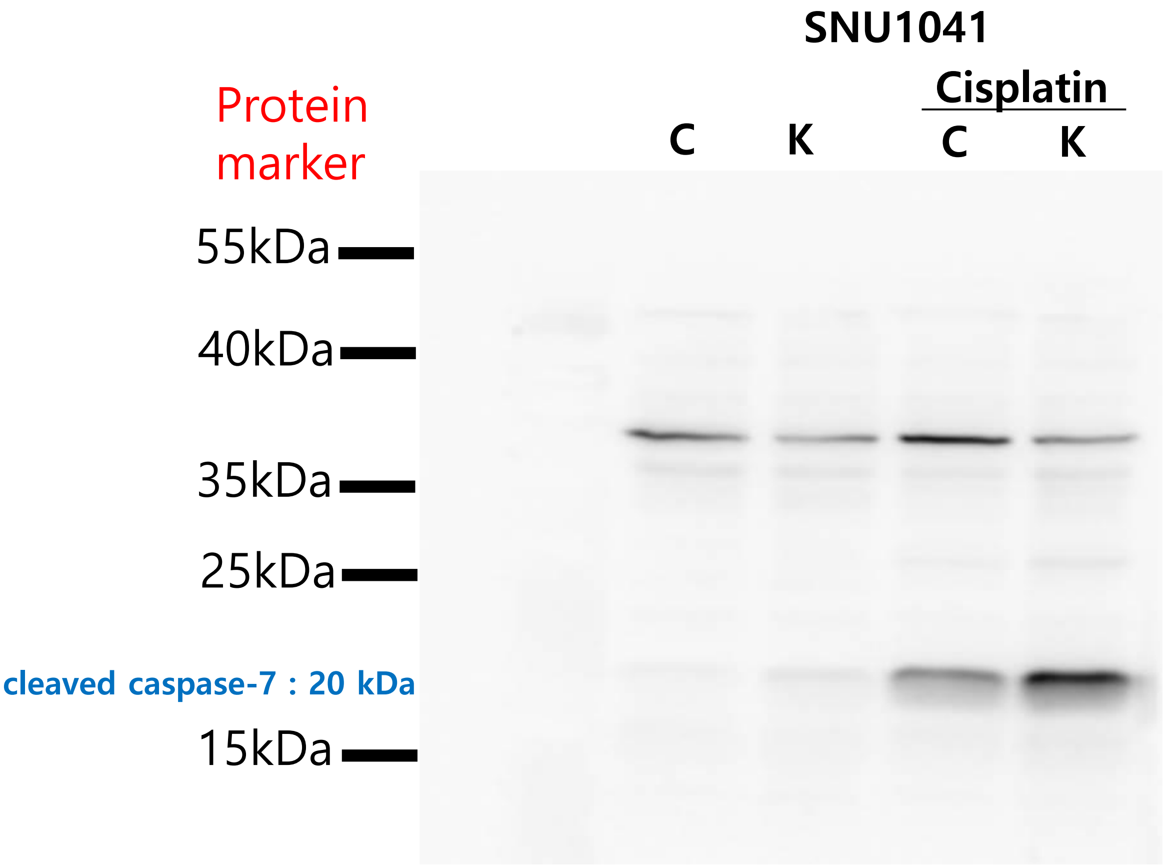

Uncropped whole western blot showing expression level of cleaved caspase-7 on SNU-1041 cells with cisplatin of figure 7 is showed with molecular weight markers.

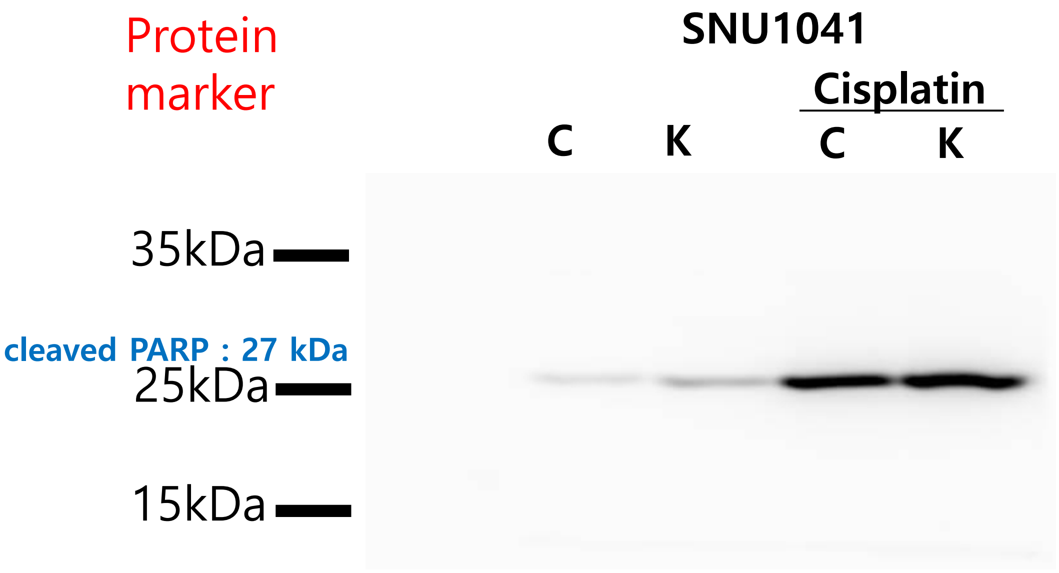

Uncropped whole western blot showing expression level of cleaved PARP on SNU-1041 cells with cisplatin of figure 7 is showed with molecular weight markers.

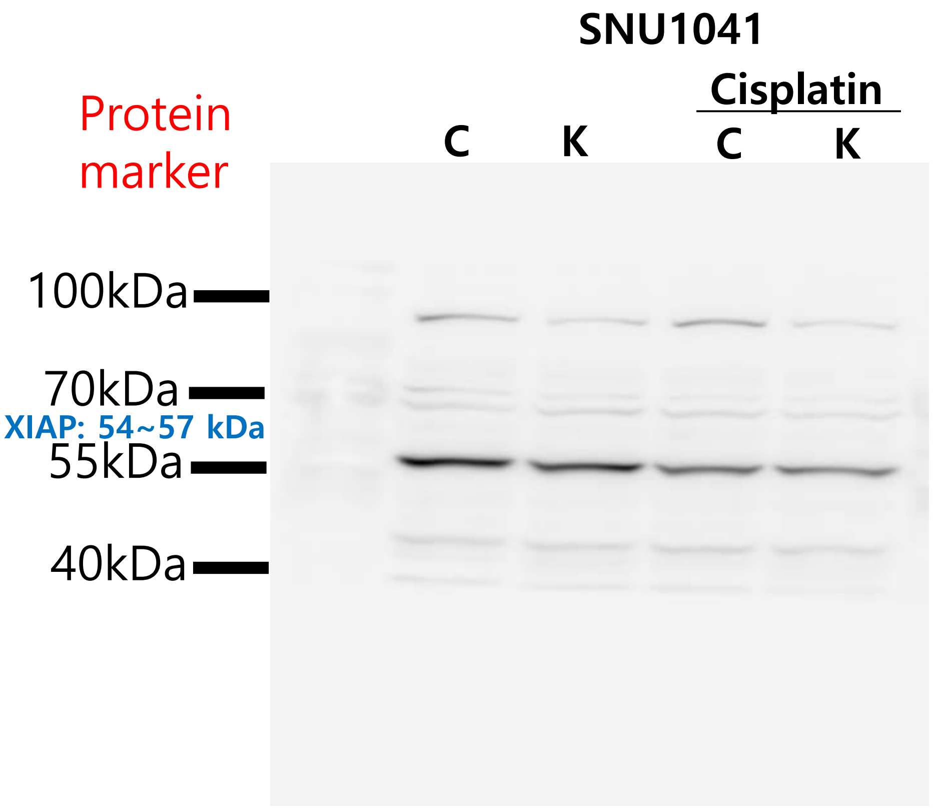

Uncropped whole western blot showing expression level of XIAP on SNU-1041 cells with cisplatin of figure 7 is showed with molecular weight markers.

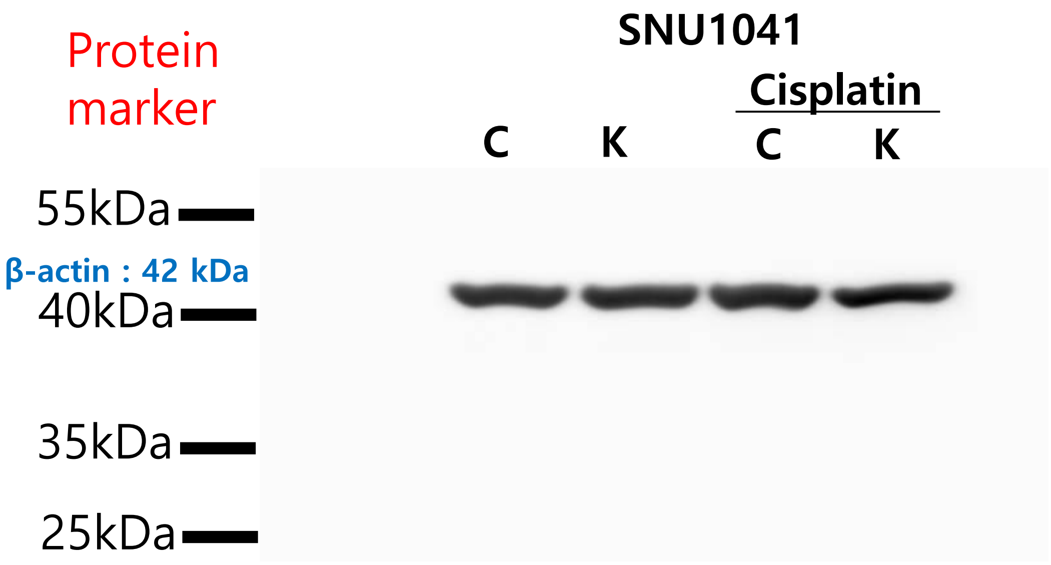

Uncropped whole western blot showing expression level of β-actin on SNU-1041 cells with cisplatin of figure 7 is showed with molecular weight markers.

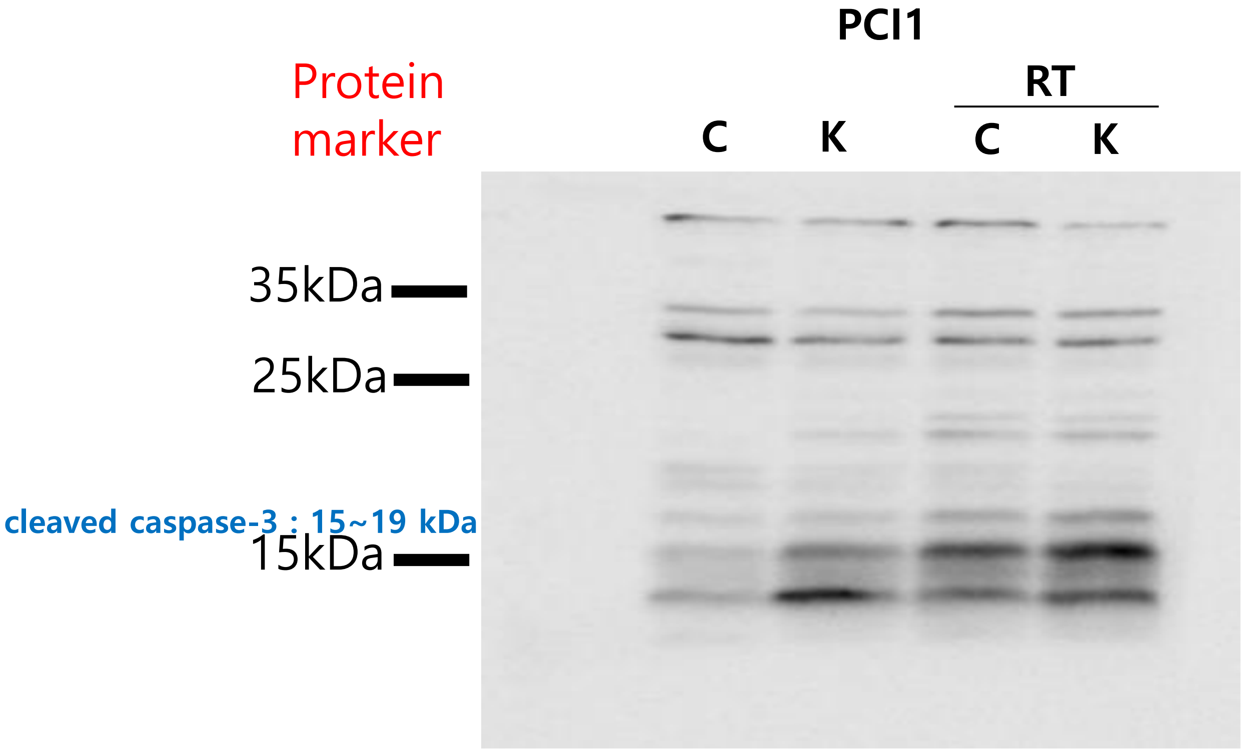

Uncropped whole western blot showing expression level of cleaved caspase-3 on PCl1 cells with RT of figure 7 is showed with molecular weight markers.

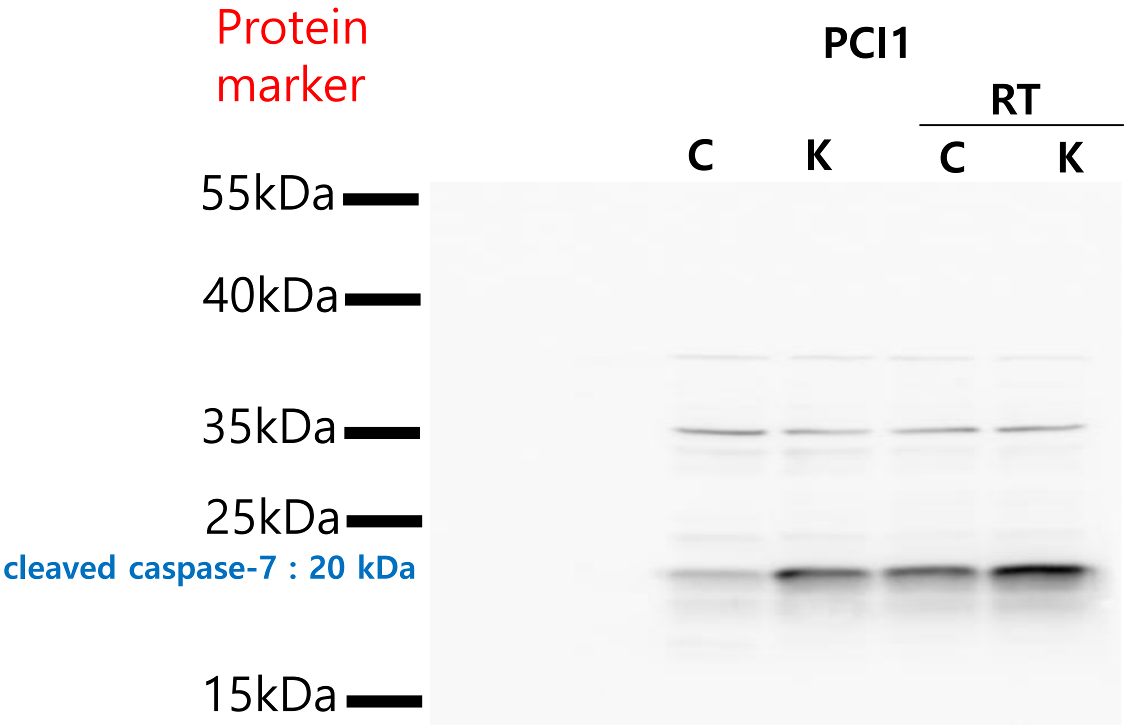

Uncropped whole western blot showing expression level of cleaved caspase-7 on PCl1 cells with RT of figure 7 is showed with molecular weight markers.

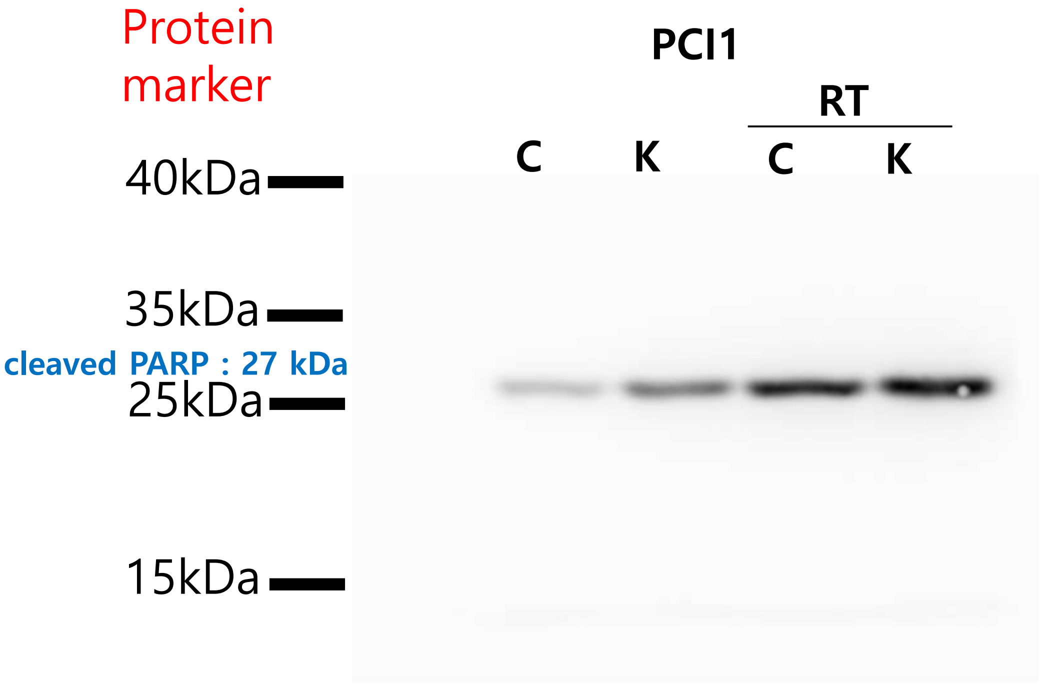

Uncropped whole western blot showing expression level of cleaved PARP on PCl1 cells with RT of figure 7 is showed with molecular weight markers.

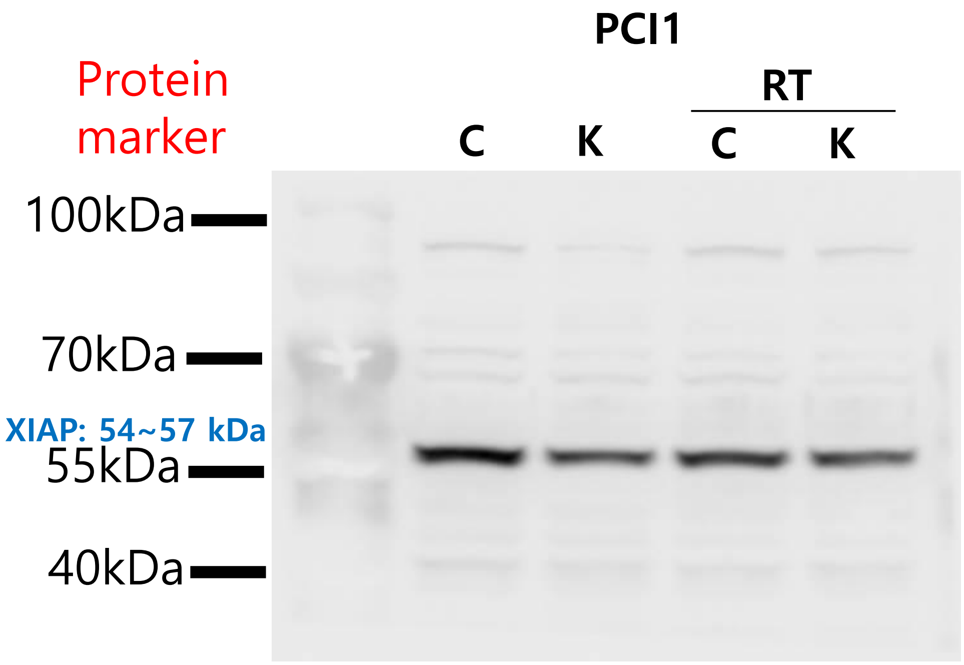

Uncropped whole western blot showing expression level of XIAP on PCl1 cells with RT of figure 7 is showed with molecular weight markers.

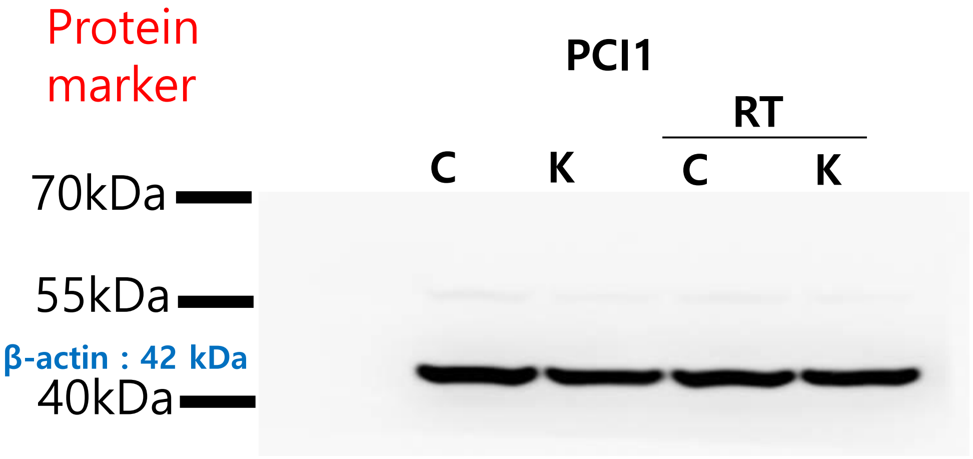

Uncropped whole western blot showing expression level of β-actin on PCl1 cells with RT of figure 7 is showed with molecular weight markers.

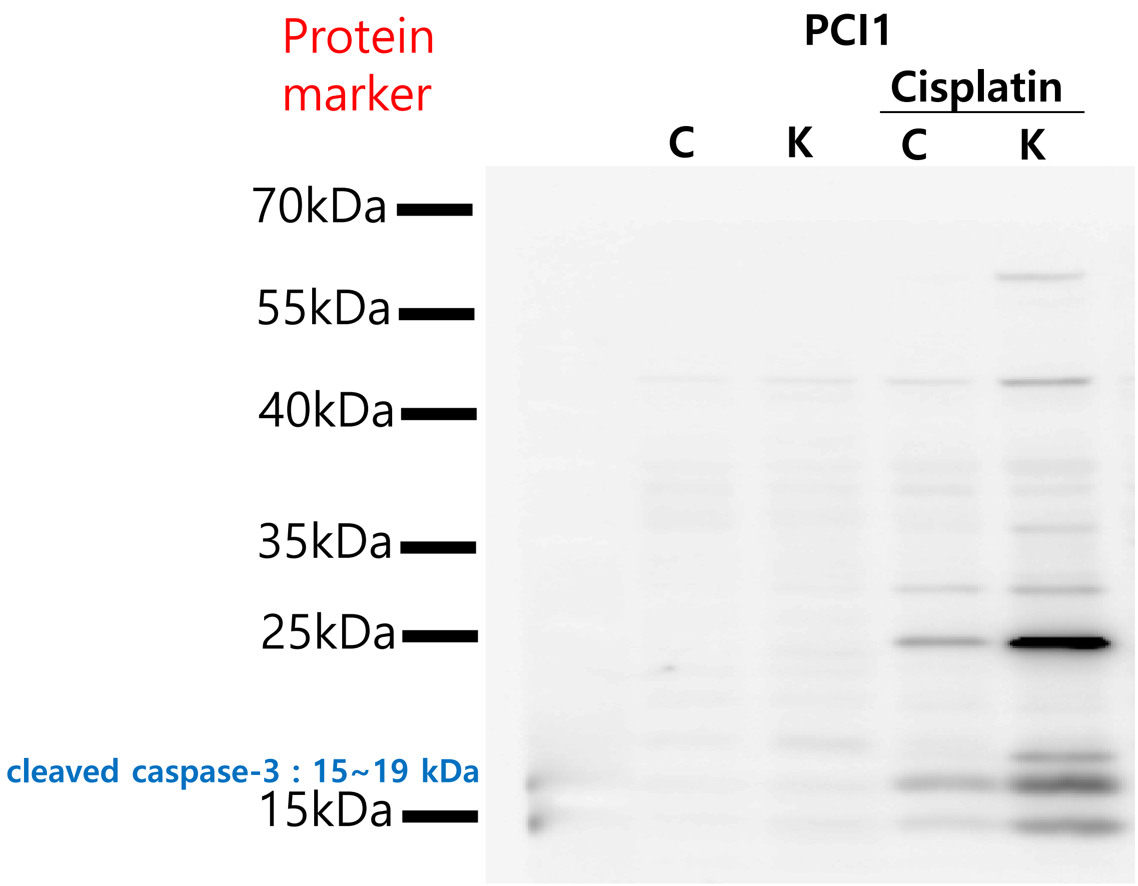

Uncropped whole western blot showing expression level of cleaved caspase-3 on PCl1 cells with cisplatin of figure 7 is showed with molecular weight markers.

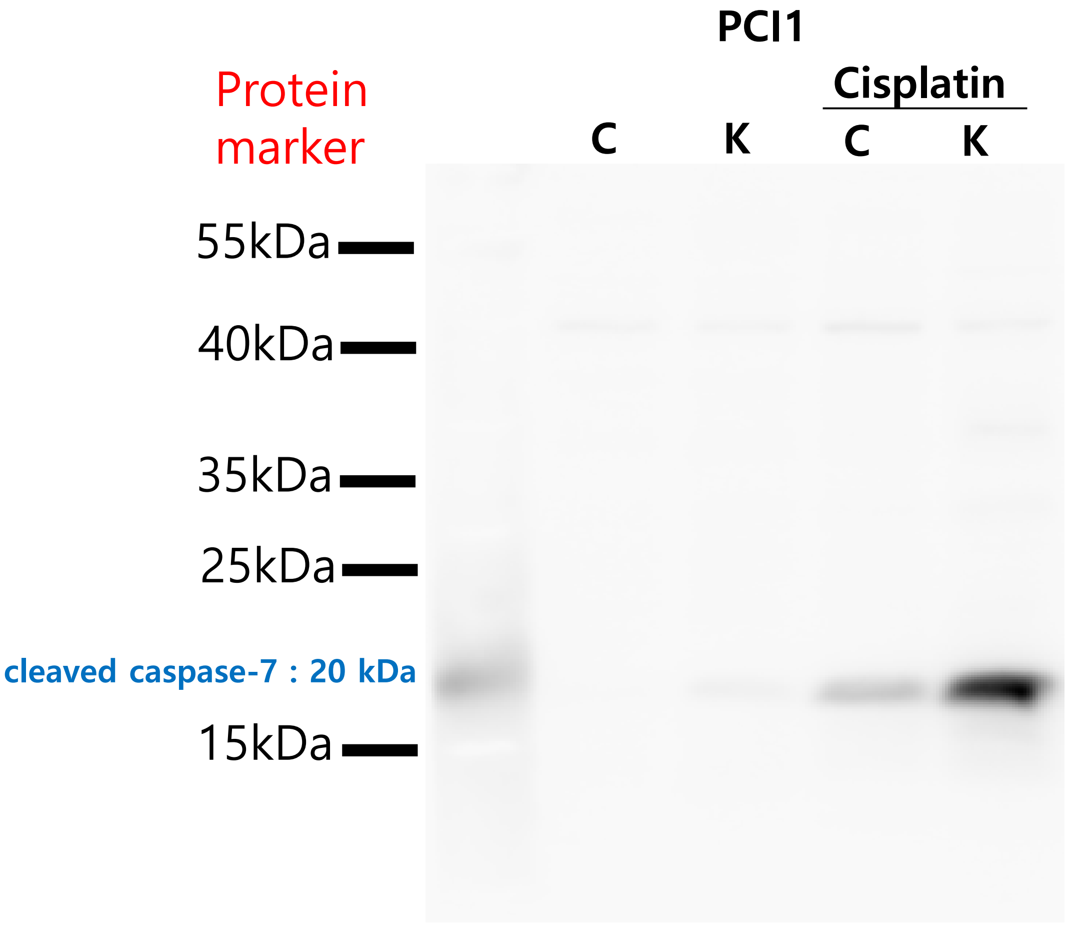

Uncropped whole western blot showing expression level of cleaved caspase-7 on PCl1 cells with cisplatin of figure 7 is showed with molecular weight markers.

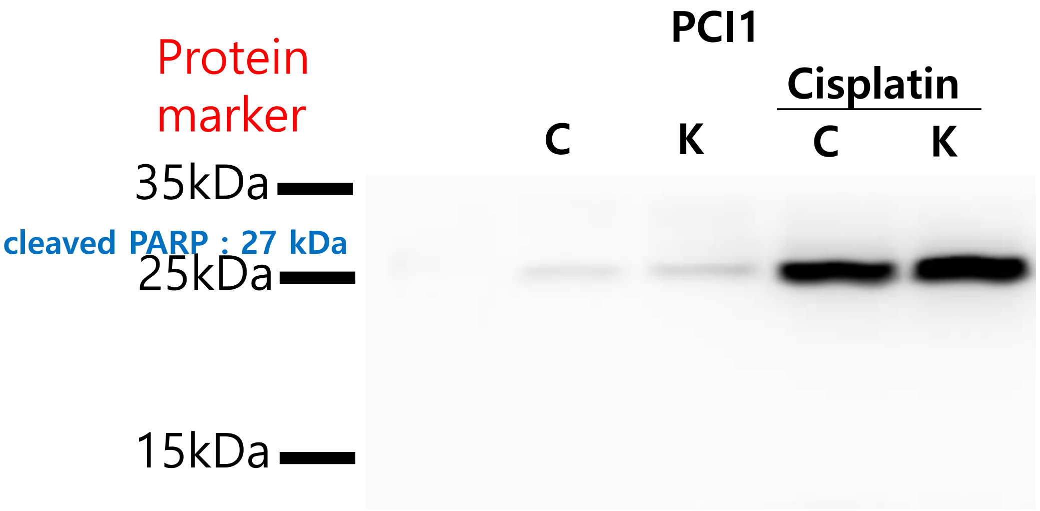

Uncropped whole western blot showing expression level of cleaved PARP on PCl1 cells with cisplatin of figure 7 is showed with molecular weight markers.

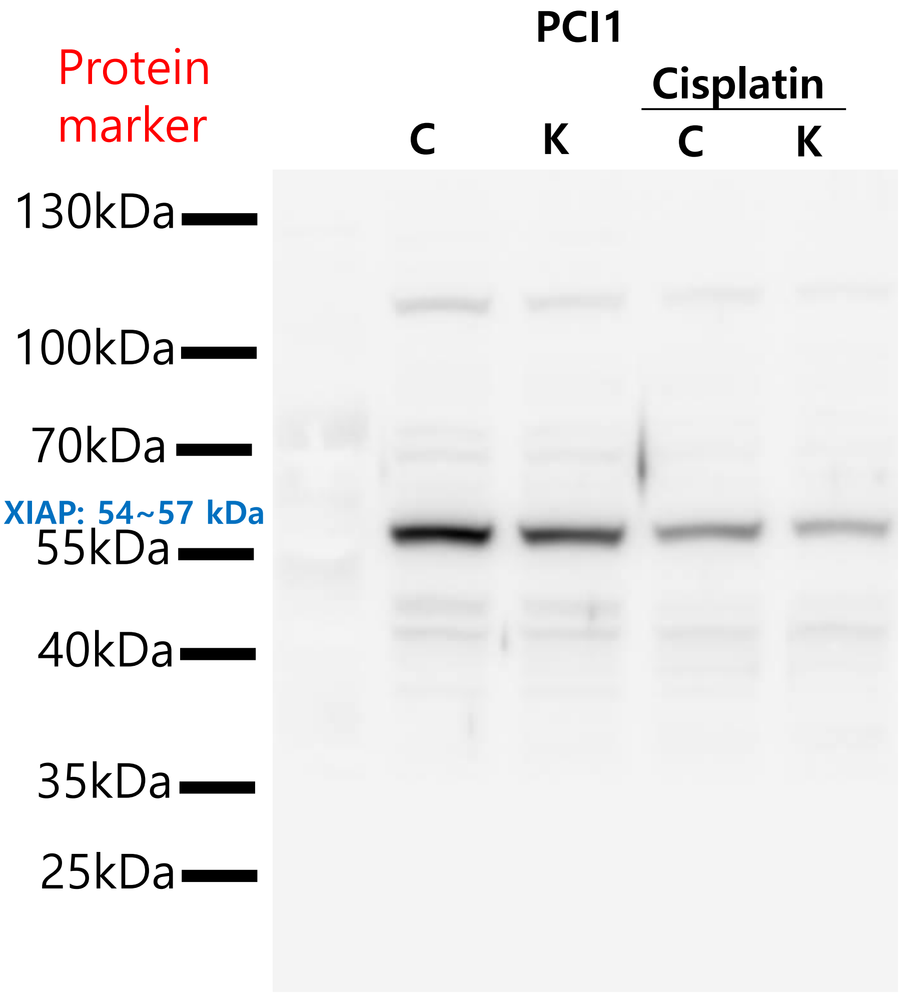

Uncropped whole western blot showing expression level of XIAP on PCl1 cells with cisplatin of figure 7 is showed with molecular weight markers.

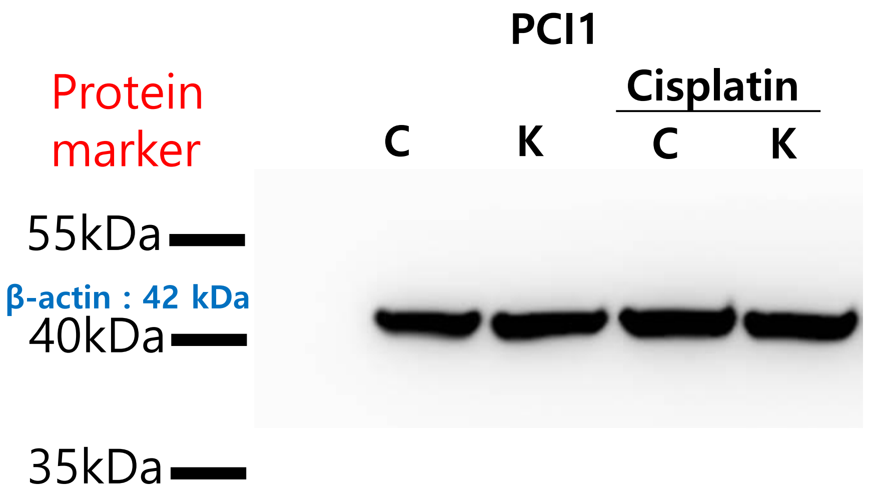

Uncropped whole western blot showing expression level of β-actin on PCl1 cells with cisplatin of figure 7 is showed with molecular weight markers.
